# Acid-Base Equilibrium and Dielectric Environment Regulate Charge in Supramolecular Nanofibers

**DOI:** 10.3389/fchem.2022.852164

**Published:** 2022-03-16

**Authors:** Rikkert J. Nap, Baofu Qiao, Liam C. Palmer, Samuel I. Stupp, Monica Olvera de la Cruz, Igal Szleifer

**Affiliations:** ^1^ Department of Biomedical Engineering, Northwestern University, Evanston, IL, United States; ^2^ Chemistry of Life Processes Institute, Northwestern University, Evanston, IL, United States; ^3^ Department of Materials Science and Engineering, Northwestern University, Evanston, IL, United States; ^4^ Simpson Querrey Institute for BioNanotechnology, Northwestern University, Chicago, IL, United States; ^5^ Department of Chemistry, Northwestern University, Evanston, IL, United States; ^6^ Department of Medicine, Northwestern University, Chicago, IL, United States; ^7^ Department of Chemical and Biological Engineering, Northwestern University, Evanston, IL, United States; ^8^ Department of Physics and Astronomy, Northwestern University, Evanston, IL, United States; ^9^ Center for Computation and Theory of Soft Materials, Northwestern University, Evanston, IL, United States

**Keywords:** peptide amphiphiles, charge regulation, ion condensation, theory, dielectric constant

## Abstract

Peptide amphiphiles are a class of molecules that can self-assemble into a variety of supramolecular structures, including high-aspect-ratio nanofibers. It is challenging to model and predict the charges in these supramolecular nanofibers because the ionization state of the peptides are not fixed but liable to change due to the acid-base equilibrium that is coupled to the structural organization of the peptide amphiphile molecules. Here, we have developed a theoretical model to describe and predict the amount of charge found on self-assembled peptide amphiphiles as a function of pH and ion concentration. In particular, we computed the amount of charge of peptide amphiphiles nanofibers with the sequence *C*_16_ − *V*_2_ *A*_2_ *E*_2_. In our theoretical formulation, we consider charge regulation of the carboxylic acid groups, which involves the acid-base chemical equilibrium of the glutamic acid residues and the possibility of ion condensation. The charge regulation is coupled with the local dielectric environment by allowing for a varying dielectric constant that also includes a position-dependent electrostatic solvation energy for the charged species. We find that the charges on the glutamic acid residues of the peptide amphiphile nanofiber are much lower than the same functional group in aqueous solution. There is a strong coupling between the charging via the acid-base equilibrium and the local dielectric environment. Our model predicts a much lower degree of deprotonation for a position-dependent relative dielectric constant compared to a constant dielectric background. Furthermore, the shape and size of the electrostatic potential as well as the counterion distribution are quantitatively and qualitatively different. These results indicate that an accurate model of peptide amphiphile self-assembly must take into account charge regulation of acidic groups through acid–base equilibria and ion condensation, as well as coupling to the local dielectric environment.

## 1 Introduction

The Stupp laboratory has developed a class of peptide amphiphiles that spontaneously form high-aspect-ratio, filamentous nanostructures in water (Hartgerink et al., 2001). These peptide amphiphile molecules typically consist of a hydrophobic hydrocarbon tail conjugated to a sequence of amino acids with the propensity to form *β*-sheet hydrogen bonds and charged amino acids for solubility. The headgroups of these peptides typically contain amino acid residues with weakly acidic or weakly basic side chains (Hendricks et al., 2017). The competition between the hydrophobic interactions of the alkyl chain, the packing of the alkyl chain and headgroup, electrostatic interactions originated from the chargeable amino acid residues as well as the ability of the amino acid residues to form hydrogen bonds, causes peptide amphiphiles to self-assemble into a variety of nanostructures such as fibers, ribbons, bilayers, and micelles (Hendricks et al., 2017).

The self-assembly of peptide amphiphile (PA) molecules is controlled by the alkyl chain length, the formation of *β*-sheet hydrogen bonds and headgroup size and charge (Dehsorkhi et al., 2014). While the influence of the alkyl chain length and headgroup size on the self-assemble is relatively well understood for simple surfactants (Israelachvili, 2011), a fundamental understanding and control of the charge found in PA nanostructures are still lacking, particularly when the charge is not fixed but originates from amino acids with variable ionization states like glutamic acid (Deiss-Yehiely et al., 2017; Ortony et al., 2017). However, determining and modeling the effective charge density is crucial for understanding the PA self-assembly behavior and to optimize the sample processing conditions for a particular application. Furthermore, recent studies have shown that the molecular dynamics is critical for the PA nanostructures to form hierarchical structures (Freeman et al., 2018; Stupp et al., 2020; Wester et al., 2020) and to optimize biological signaling (Álvarez et al., 2021).

The filamentous peptide amphiphile nanostructures have been shown to mimic the extracellular matrix and have shown great potential for drug delivery and the regeneration of many different tissues (Matson and Stupp, 2012; Sato et al., 2018). Beyond their application as biomaterials, peptide amphiphiles have also been explored in material science applications and have been used to construct anisotropic actuating materials (Chin et al., 2018; Li et al., 2020). Consequently, there is an increasing number of experimental as well as theoretical studies into properties, behavior, and applications of peptide amphiphiles.

Experimentally, it is difficult to directly measure the amount of charge found in self-assembled peptide amphiphiles, since experimental techniques rely on indirect measurements. For example, *ζ*-potential measurements, a common tool employed in colloid chemistry, measures the electrophoretic mobility which can be used to estimate the electrostatic surface potential and charge. Also this method is best used for spherical particles and can it be more challenging to interpret for filamentous/fibrous structures. Ion counting or inductively coupled plasma mass spectroscopy (ICPMS) experiments (Gebala et al., 2015), can determine the number of condensed counterions and counterions contained in the cloud that surrounds a charged molecule or particle. *ζ*-Potential and ICMPS measurements provide an indirect indication of the strength of the electrostatic potential and charge of the charged moiety.

Small-angle X-ray scattering (SAXS) has been used to study supramolecular nanofibers (Bitton et al., 2005; Moyer et al., 2014; Kornmueller et al., 2016; Hendricks et al., 2017; Kornmueller et al., 2017). Anomalous small angle X-ray scattering (ASAXS) has emerged as a useful tool to determine the spatial distribution of ions surrounding charged macromolecules or self-assembled aggregates (Ballauff and Jusufi, 2006; Sztucki et al., 2012). This is done by careful subtraction of the scattered intensity profiles in the small-angle region at energies near and far away from an absorption edge of a targeted element such as rubidium or bromine. This technique has been used to determine the counterion distribution surrounding DNA (Das et al., 2003; Andresen et al., 2004), DNA-capped proteins (Krishnamoorthy et al., 2018) and spherical and cylindrical nanoparticles (Dingenouts et al., 2004; Patel et al., 2004).

Titration offers another method to establish (indirectly) the charge of self-assembling peptide amphiphiles. There are few titration studies of peptide amphiphile fibers and micelles (Toksoz et al., 2011; Buettner et al., 2017; Gao et al., 2017), due to bundling of the nanofibers at low or high pH values depending on whether the PA side chains are acidic or basic. Likewise, the possibility of morphological transitions make these titration experiments difficult. The interpretation of the titration curves is also complicated since a peptide amphiphile molecule can consists of multiple interacting amino acids. Furthermore the peptide amphiphile molecules are densely packed within the nanostructures and their ionizable side chains exist in a range of different chemical environments. Therefore (simple) relations, like the Henderson-Hasselbach (Atkins, 1998) equation, which estimates the amount of charge of a simple monoprotic acid in dilute solution, can not be applied to titration curves of self-assembled peptide amphiphiles.

Theoretically, molecular dynamics (MD) simulations have provided many insights into the self-assembly of peptide amphiphiles (Velichko et al., 2008; Lee et al., 2011, 2012). While MD simulations include many molecular details, they generally impose a fixed charge distribution and do not consider the possibility of dynamic chemical equilibria between protonation and deprotonation of acids. Acid-base chemical equilibrium can be introduced in MD simulations using constant-pH or reaction ensemble simulations (Donnini et al., 2011; Landsgesell et al., 2019), but this is difficult and costly to implement except in small, relatively simple systems (Donnini et al., 2011; Cote et al., 2014). Nanofibers, which involve many PA molecules, are therefore typically modeled with coarse-grained simulations, which impose fixed charges and other constraints and assumptions to predict the structure formation (Lee et al., 2011; Lee et al., 2012). Since self-assembling peptide amphiphiles often include amino acid side chains with weak acids like glutamic acid or aspartic acid (or weak bases like lysine), the extent of deprotonation or ionization is highly pH dependent. Another limitation is that MD simulations, due to their time-consuming nature, are not practical for systematic variation of parameters like salt concentration and pH.

Here, we present a theoretical model to describe the charge of a PA-nanofiber that includes the chemical equilibrium between the protonated, deprotonated, and ion-condensed states of the amino acid residue and the effect of dielectric environment and solvation. Importantly, the theory does not assume the charged state of the acid residues of the peptide but rather predicts the position-dependent state of charge. The theory is based on a molecular statistical thermodynamic approach that has previously been developed to predict thermodynamic and structural properties of end-tethered weak polyelectrolytes, which are polymers whose degree of deprotonation is not fixed but can change depending on environmental conditions including pH and ionic concentration (Solveyra et al., 2020). Predictions of this theory on the height of poly-acrylic acid brushes and the charged state of acid ligated gold nanoparticles were in good agreement with experimental observations (Gong et al., 2007; Wang et al., 2011). Likewise, a similar approach was used to investigate the effect of solution conditions on the charge regulation of bacteriophage capsids (Nap et al., 2014a) and the absorption of acidic ligands to quantum dots (Westmoreland et al., 2019). Also noteworthy is a recent study by Tagliazucchi et al. in which the molecular theory was used to study the self-assembly of neutral and chargeable peptide amphiphiles (Zaldivar et al., 2018; Zaldivar et al., 2019). However, ion-condensation and effects related to the electrostatic solvation free energy were not considered.

The objective of this theoretical study is to gain more insight into the effect that pH and other solution conditions, like type and amount of counterions, have on the amount of charge in the PA-nanofiber. To this end we consider a peptide amphiphiles with sequence *C*_16_ − *V*_2_ *A*_2_ *E*_2_, which is well characterized experimentally (Goldberger et al., 2011; Ortony et al., 2014).

One of the major findings is that there is a very strong coupling between the acid-base equilibrium and the local dielectric environment, which results in a much lower degree of deprotonation of the glutamic acid groups of the PA-nanofiber compared to the degree of deprotonation of the same acid in solution. Assuming a constant dielectric background, a common approximation employed in theoretical descriptions of colloids, polyelectrolyte solutions, and end-tethered polyelectrolyte layers, results in qualitatively and quantitatively very different degrees of deprotonation of the acid groups as compared to considering a position-dependent dielectric constant plus electrostatic solvation energy.

The paper is organized as follows. First, we present the theoretical approach. This is followed by a presentation and discussion of the results. Finally, we end with a summary, concluding remarks, and indicate potential future directions.

## 2 Theoretical Approach

**FIGURE 1:**
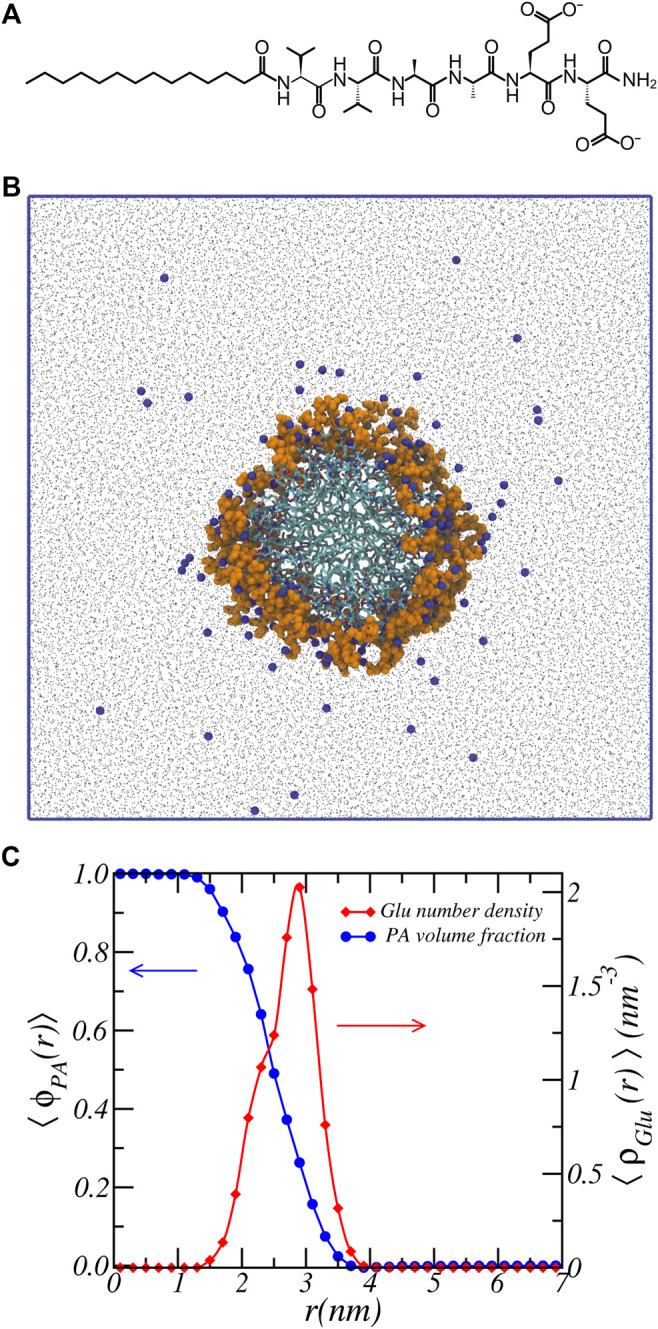
Atomistic simulation snapshot and density profiles. **(A)** chemical structure of *C*_16_ − *V*_2_ *A*_2_ *E*_2_. **(B)** Snapshot of the last simulation configuration. *C*_16_ − *V*_2_ *A*_2_ *E*_2_ nanofiber is highlighted at the center, with glutamic acid residue colored in orange and hydrogen atoms omitted for display. *Na* ^+^ ions are represented by blue beads, and water by black dots. Blue solid lines denote the simulation box. Aggregation number is 17.3 ± 0.1 *PA*/*nm*. **(C)** Volume fraction of PA-fiber and number density of Glu units as a function of radial cylindrical coordinate.

Here we consider *C*_16_ − *V*_2_ *A*_2_ *E*_2_, a well studied peptide amphiphile, consists of an aliphatic tail of 16 hydrocarbons linked to a peptide sequence of two valines, two alanines, and two glutamic acids, which forms a nanofiber. Figure 1 shows a “schematic” of the PA-nanofiber. The self-assembling PA-nanofiber is in contact with an aqueous solution. The reservoir is characterized by a given pH and contains either NaCl to mimic the high salt of biological media or RbCl since Rb^+^ provides a higher atomic number with better contrast for X-ray studies like anomalous small-angle X-ray scattering (ASAXS) (Ballauff and Jusufi, 2006). In this model, we assume complete dissociation of the salt ions. The pH is adjusted by adding either HCl or RbOH. In case of an aqueous solution that contains NaCl salt, either HCl or NaOH is added.

The carboxylic acid of the glutamic amino acid residue can be found either in a deprotonated state (A^−^), a protonated state (AH) or a state in which the acid is condensed with either a Rb^+^ or a Na^+^ counterion, which are denoted by ARb and ANa, respectively.

Following chemical reactions are included

$$\begin{array}{ccc} & AH⇌A^{-}+H^{+}, & \end{array}$$ (1)

$$\begin{array}{ccc} & A^{-}+Na^{+}⇌ANa, & \end{array}$$ (2)

$$\begin{array}{ccc} & A^{-}+Rb^{+}⇌ARb. & \end{array}$$ (3)

Considering both Rb^+^ counterions as well as Na^+^ ions allows us to investigate how different monovalent ions affect the protonation state of PAs.

The free energy describing one PA-nanofiber in contact with an electrolyte solution has a number of distinct contributions, which can be summarized as follows

$$F=-TS_{\text{conf,PA}}-TS_{\text{mix,PA}}+F_{\text{hb,PA}}+E_{\text{VdW}}-TS_{\text{mix}}+F_{\text{chem}}+F_{\text{elect}}+E_{\text{elect, solv}}+E_{\text{rep}}.$$ (4)

The first four contributions are related to the conformational and translational entropy of the PA molecules, the van der Waals or hydrophobic interactions among the hydrocarbons and amino acids and the possibility to from hydrogen bonds between the amino-acids residues, respectively. The following four contributions are stemming from the mixing entropy of the mobile ions and solvent (*S*_mix_), the acid-base chemical equilibrium of the acid group of the glutamic acid and ion condensation (*F*_chem_), the electrostatic interaction energy (*F*_elect_), and the electrostatic solvation energy of the charged species (*F*_elect, solv_). The last term, *E*_rep_ accounts for the steric repulsions among all molecular species. We shall invoke a simplifying approximation. Therefore, we shall only present the pertinent terms and relegate the presentation of the complete theory to the supporting material.

The − *TS*_mix_ in the free energy corresponds to the mixing or translational entropy per unit length of the PA-nanofiber of the solvent (water) and mobile ionic species

$$-\frac{S_{mix}}{k_{B}L}=\underset{k}{\sum }\int drG\left(r\right)\rho_{k}\left(r\right)\left(\ln\left(\rho_{k}\left(r\right)v_{w}\right)-1\right).$$ (5)

The index *k* runs over all the different types of mobile species: the water molecules, cations (Rb^+^, Na^+^), anions (Cl^−^), protons (H^+^), and hydroxide ions (OH^−^). The variable *ρ*_*k*_(*r*) corresponds to the number density of mobile species *k* and *v*_*w*_ is the volume of a water molecule, which is used as the unit of volume. We use cylindrical coordinates to reflect the symmetry of the PA-nanofiber, and assume the system to be laterally homogeneous and only explicitly anisotropic in the radial direction *r*. The function *G*(*r*)*dr* is the cylindrical volume element divided by the unit length of the PA-nanofiber and equals *G*(*r*)*dr* = *A*(*r*)*dr*/*L* = 2*πrdr*. The next term in the free energy, *F*_chem_, describes the chemical free energy associated with (de)protonation of the acid of the Glu amino acid and the ion condensation of Rb^+^ and Na^+^.

$$\begin{array}{l} \frac{F_{\text{chem}}}{k_{B}TL}=\int drG\left(r\right)\left\langle \rho_{Glu}\left(r\right)\right\rangle \left[f_{A^{-}}\left(r\right)\left(\ln f_{A^{-}}\left(r\right)+\beta \mu_{A^{-}}^{⊖}\right)+f_{AH}\left(r\right)\left(\ln f_{AH}\left(r\right)+\beta \mu_{AH}^{⊖}\right)\right.\\ +f_{ANa}\left(r\right)\left(\ln f_{ANa}\left(r\right)+\beta \mu_{ANa}^{⊖}\right)\left.\right)+\left.f_{ARb}\left(r\right)\left(\ln f_{ARb}\left(r\right)+\beta \mu_{ARb}^{⊖}\right)\right]+\\ \underset{k\in \left\{H^{+},OH^{-},Rb^{+},Na^{+}\right\}}{\sum }\beta \mu_{k}^{⊖}\int drG\left(r\right)\rho_{k}\left(r\right). \end{array}$$ (6)

Here, the $f_{A^{-}}\left(r\right)$ is the fraction of glutamic acid residues that are deprotonated at position *r*, *f*_*AH*_(*r*) is the fraction of protonated acids, and *f*_*ARb*_(*r*) and *f*_*ANa*_(*r*) are the fractions of acids that are condensed with either Rb^+^ or Na^+^. The variable $\mu_{i}^{⊖}$ corresponds to the standard chemical potential for a molecule of type *i*. The first and third terms within Eq. 6 describe the entropy of the deprotonated charged state (*A* ^−^) and protonated state (*AH*), respectively. The second and fourth terms in Eq. 6 correspond to the standard chemical potential of the charged and uncharged state, respectively. The subsequent terms have an identical meaning and pertain to the enthalpic and entropic contribution arising from the ion-condensation of Rb^+^ and Na^+^.

The seventh term, *F*_elect_, in the free energy functional, Eq. 4 describes the electrostatic contribution to the free energy (Schwinger et al., 1998) and is given by

$$\frac{F_{\text{elect}}}{L}=\int drG\left(r\right)\left[\left\langle \rho_{q}\left(r\right)\right\rangle \psi \left(r\right)+\frac{1}{2}\epsilon_{0}\epsilon_{r}\left(r\right){\left(\nabla_{r}\psi \left(r\right)\right)}^{2}\right].$$ (7)

Here, *ψ*(*r*) is the electrostatic potential and ⟨*ρ*_*q*_(*r*)⟩ is the total charge density. The total charge density is the sum of the charge density of all charged mobile ions and the charge density of the amino-acid residues

$$\langle \rho_{q}\left(r\right)\rangle =\underset{i}{\sum }ez_{i}\rho_{i}\left(r\right)+ez_{A^{-}}f_{A^{-}}\left(r\right)\langle \rho_{Glu}\left(r\right)\rangle .$$ (8)

Here, the summation runs over all charged mobile ions Rb^+^, Na^+^, Cl^−^, H^+^, OH^−^ with *z*_*i*_ corresponding to their valency. *e* is the unit of charge. The second term is the number density of amino-acid residues that are deprotonated: $\left\langle \rho_{A^{-}}\left(r\right)\right\rangle =f_{A^{-}}\left(r\right)\left\langle \rho_{Glu}\left(r\right)\right\rangle$ .

In the electrostatic functional, *ϵ*_0_ and *ϵ*_*r*_(*r*) correspond to the dielectric permittivity of vacuum and the local relative dielectric constant, respectively. Variation of the above functional with respect to the electrostatic potential yields the Poisson equation (Schwinger et al., 1998; Wang, 2008b). As shown by Z.-G.Wang, using field-theoretical arguments, (Wang, 2008a, 2010), consideration of a varying dielectric constant, that is having a non-uniform inhomogeneous dielectric medium, also requires the inclusion of a non-uniform electrostatic self-energy into the free energy functional. This electrostatic solvation energy (Wang and Wang, 2014) is the electrostatic energy required to transfer a charged molecule into a dielectric medium with a given position-dependent dielectric constant and is represented through a Born-type solvation energy. It is given by

$$\frac{E_{\text{elect, solv}}}{L}=\underset{k}{\sum }\int drG\left(r\right)\Delta u_{k}^{B}\left(r\right)\rho_{k}\left(r\right)\;\text{with}\Delta u_{k}^{B}\left(r\right)=\frac{z_{k}^{2}e^{2}}{8\pi \epsilon_{0}a_{k}}\left(\frac{1}{\epsilon_{r}\left(r\right)}-\frac{1}{\epsilon_{w}}\right).$$ (9)

For every ion of type *k*, *a*_*k*_ corresponds to the radius and *z*_*k*_ represents its valence. In order to describe the dielectric medium we need to provide a constitutive equation for the relative dielectric constant. We assume that the relative dielectric function of the medium is the volume-weighted average of the dielectric constant of water and the PA-nanofiber.

$$\epsilon_{r}\left(r\right)=\underset{i}{\sum }\epsilon_{i}\rho_{i}\left(r\right)\approx \epsilon_{PA}\langle \phi_{PA}\left(r\right)\rangle +\epsilon_{w}\left(1-\langle \phi_{PA}\left(r\right)\rangle \right)$$ (10)

Here, we ignore the effect that the local volume fractions of the ions have. The dielectric constant of water equals *ϵ*_*w*_ = 78.54, while we set the dielectric constant of the PA-fiber equal to *ϵ*_*PA*_ = 2 (oil).

Using a uniform dielectric background media with a dielectric constant equal to that of the solvent is a common approximation in classical density functional theories (DFT) and Poisson-Boltzmann approaches that describe electrolyte solutions or electrolyte solutions near interphases, because the density of the electrolytes is low (Avni et al., 2019). A uniform dielectric background media is also employed in modeling end-tethered polyelectrolyte layers and is shown to work reasonably well, even for polyelectrolyte layers with higher grafting densities (Nap et al., 2006; Nap et al., 2014b). Electrostatic solvation free energy contribution, albeit constant, also occurs in, for example, the modeling of electrolyte adsorption to liquid-liquid interphases (Kung et al., 2009). Considering that the PA-nanofiber has a very dense aliphatic core assuming a uniform dielectric constant might be questioned. Therefore, we shall consider the case of a varying dielectric constant as well as a uniform dielectric background.

The last term in Eq. 4, *E*_rep_, describe the repulsive interactions between all molecules, which, in the theory, are modeled as excluded volume interactions. These intermolecular excluded volume interactions are accounted for by assuming that the system is incompressible at every position

$$\left\langle \phi_{PA}\left(r\right)\right\rangle +\underset{k}{\sum }\phi_{k}\left(r\right)=1.$$ (11)

Here *ϕ*_*k*_(*r*) = *ρ*_*k*_(*r*)*v*_*k*_ is the volume fraction of mobile species *k* with volume *v*_*k*_ and ⟨*ϕ*_*PA*_(*r*)⟩ is the volume fraction of the PA-fiber. These volume packing constraints are enforced through the introduction of the Lagrange multipliers *π*(*r*) since these are constraints, they are not formally part of the Helmholtz free energy. The volume fraction of the PA-nanofiber is the volume weighted average of the density of all residues of the peptide amphiphiles

$$\langle \phi_{PA}\left(r\right)\rangle =\underset{i\in \left\{A^{-},AH,ARb,ANa\right\}}{\sum }\langle \rho_{Glu}\left(r\right)\rangle f_{i}\left(r\right)v_{i}+\underset{r}{\sum }\rho_{r}\left(r\right)v_{r}.$$ (12)

The first term describes the volume associated with the glutamic acid residues. Since we allow for volume changes upon ion-condensation to the glutamic acids, a summation over all charged states of the glutamic acid residues is required. The second contribution in Eq. 12 represents all other residues and the hydrocarbon tail.

The total free energy is minimized with respect to the number density of all species, *ρ*_*i*_(*r*), the fraction of the charged states, *f*_*k*_(*r*), and varied with respect to the electrostatic potential, *ψ*(*r*), under the constraints of incompressibility and the fact that the system is in contact with a bath of cations, anions, protons, and hydroxide ions. Thus, the proper thermodynamic potential is the semi-grand potential (Nap et al., 2006; Nap et al., 2014b): Ω = *F* − *∑*_*i*_ *μ*_*i*_ *N*_*i*_, with *N*_*i*_ denoting the total number of particles of species *i*.

It is important to note that the complete theory, as formulated in the supporting material, is also dependent on the volume fraction of the PA-nanofiber, ⟨*ϕ*_*PA*_(*r*)⟩, and the Glu number density, ⟨*ρ*_*Glu*_(*r*)⟩. Meaning the total free energy includes the conformational energy and van der Waals interactions of the PA as well. However, due to technical challenges, we have not been unable to solve the complete molecular theory for appropriate large PA aggregation numbers yet. At these conditions the solvent is expelled from the aliphatic core and it becomes numerical challenging to satisfy the packing constraint. Hence we invoked an additional approximation. Namely, we imposed the volume fraction distribution of the PA-nanofiber and the distribution of the Glu number density. Similar approximations have been employed to model the counterion distribution around Au-NPs coated with DNA (Kewalramani et al., 2013) and as well as protein-nucleic acid conjugates (Krishnamoorthy et al., 2018).

To obtain a reasonable estimate of the volume fraction of the PA-nanofiber and the Glu number density, we use predictions from atomistic MD simulations. Technically, the simulations provide the number density of the water $\left(\rho_{w}^{0}\left(r\right)\right)$ from which we can obtain the volume fraction of the PA-nanofiber needed as input for the theory. Ignoring the volume contributions from the ions, the volume fraction of the PA-nanofiber is then simply given as $\left\langle \phi_{PA}^{0}\left(r\right)\right\rangle =1-\rho_{w}^{0}\left(r\right)v_{w}$ . Because we allow for volume changes in the ion condensation reactions, we take the volume fraction of the PA-nanofiber from the simulation to correspond to the deprotonated state of glutamic acid. Consequently the total volume fraction of the PA-nanofiber is given by

$$\left\langle \phi_{PA}\left(r\right)\right\rangle =\left\langle \phi_{PA}^{0}\left(r\right)\right\rangle +\underset{i\in \left\{AH,ARb,ANa\right\}}{\sum }\left\langle \rho_{Glu}\left(r\right)\right\rangle f_{i}\left(r\right)\left(v_{i}-v_{A^{-}}\right).$$ (13)

Given the volume fraction profile and distribution of glutamic acid residues, we can compute using our theory the amount of charge of the Glu residues on the PA-nanofiber as well as the distribution of ions and water molecules and the electrostatic potential.

Finally, there has been considerable amount of theoretical research on charge regulation on flat surfaces (Ninham and Parsegian, 1971; Behrens and Borkovec, 1999; Boon and van Roij, 2011) and colloidal particles (Carnie et al., 1994; Stankovich and Carnie, 1996; Pericet-Camara et al., 2004; Frydel et al., 2007; Popa et al., 2010a; Popa et al., 2010b; Markovich et al., 2017; Avni et al., 2019). These are mostly formulated within the context of Poisson–Boltzmann theory, where the surface charge is either imposed to obey the acid-base equilibrium based on its solution equilibrium constant or by invoking the so-called the constant regulation approximation, (Behrens and Borkovec, 1999; Pericet-Camara et al., 2004; Popa et al., 2010a), that involves an unknown adjustable parameter. The approach presented here is, however, different. First, we consider a distribution of chargeable sites, i.e., there is a “diffusive” interface instead of a flat, impenetrable surface. Secondly, we do not impose the chemical state instead it follows from the free energy minimization. Thirdly, we considered a varying dielectric constant and electrostatic solvation energy. Common Poisson-Boltzmann based theories usually assume a fixed dielectric constant and only involve the translational entropy of the ions (*F*_mix_) and electrostatic interactions (*F*_elect_). The other terms for the free energy are usually not considered, especially the ones describing the chemical equilibrium of the acid groups, the ion-condensation of the ions and the effect of the dielectric environment as well as the excluded volume repulsion or finite size of all molecules-including water. Thus, unlike traditional Poisson-Boltzmann approaches, the current model includes the effect of charge regulation and dielectric environment as well as the type and size of ions.

Minimization of the free energy yields the following expression for the local volume fraction of the solvent

$$\phi_{w}\left(r\right)=\rho_{w}\left(r\right)v_{w}=\exp\left(-\beta \pi \left(r\right)v_{w}\right),$$ (14)

while the density of the ions reads

$$\rho_{\gamma }\left(r\right)=\frac{1}{v_{w}}\exp\left(\beta \mu_{\gamma }-\beta \mu_{\gamma }^{⊖}-\beta \pi \left(r\right)v_{\gamma }-\beta \psi \left(r\right)z_{\gamma }e\right)\exp\left(-\beta \Delta u_{k}^{B}\left(r\right)\right).$$ (15)

Observe that the Lagrange multiplier, *π*(*r*), can be interpreted as the lateral osmotic pressure. Also, notice that the chemical potential of water is not specified explicitly because the incompressibility constraint reduces the number of independent thermodynamic variables. Therefore, the chemical potential, *μ*_*γ*_, is in reality an exchange chemical potential, i.e., the difference between the chemical potential of the molecule of type *γ* and that of water. Likewise, the charge neutrality and the water self-dissociation equilibrium further reduce the number of independent thermodynamic variables. Values of the exchange chemical potentials for all species can be obtained by relating them to their reservoir concentrations: $\rho_{\gamma }^{bulk}v_{w}=\exp\left(\beta \mu_{\gamma }^{⊖}-\beta \mu_{\gamma }-\beta \pi^{bulk}v_{\gamma }\right)$ , with $\Delta u_{k}^{B,bulk}=0$ (Eq. 9) and *ψ* ^*bulk*^ = 0 (Eq. 16) (Nap et al., 2006; Nap et al., 2017).

Functional variation of the free energy with respect to the electrostatic potential yields the Poisson equation and its boundary conditions

$$-\epsilon_{0}\frac{1}{r}\frac{d}{dr}\left(\epsilon_{r}\left(r\right)r\frac{d\psi \left(r\right)}{dr}\right)=\left\langle \rho_{q}\left(r\right)\right\rangle ;{\left.-\epsilon_{0}\epsilon_{r}\left(r\right)\frac{d\psi \left(r\right)}{dr}\right|}_{r=0}=0;\underset{r\to \infty }{\lim}\psi \left(r\right)=0.$$ (16)

Minimization of the free energy with respect to the different states of the Glu residue, $f_{A^{-}}\left(r\right)$ , *f*_*AH*_(*r*), *f*_*ANa*_(*r*), and *f*_*ARb*_(*r*) results in following set of ‘chemical reaction’ equations

$$\frac{f_{A^{-}}\left(r\right)}{f_{AH}\left(r\right)}=e^{-\beta \Delta G_{AH}^{⊖}}e^{-\beta \Delta G_{AH}^{solv}\left(r\right)}\frac{e^{-\beta \pi \left(r\right)\Delta v_{AH}}}{\rho_{H^{+}}\left(r\right)v_{w}},$$ (17)

$$\frac{f_{A^{-}}\left(r\right)}{f_{ANa}\left(r\right)}=e^{-\beta \Delta G_{ANa}^{⊖}}e^{-\beta \Delta G_{ANa}^{solv}\left(r\right)}\frac{e^{-\beta \pi \left(r\right)\Delta v_{ANa}}}{\rho_{Na^{+}}\left(r\right)v_{w}},$$ (18)

$$\frac{f_{A^{-}}\left(r\right)}{f_{ARb}\left(r\right)}=e^{-\beta \Delta G_{ARb}^{⊖}}e^{-\beta \Delta G_{ARb}^{solv}\left(r\right)}\frac{e^{-\beta \pi \left(r\right)\Delta v_{ARb}}}{\rho_{Rb^{+}}\left(r\right)v_{w}}.$$ (19)

The variable $\Delta G_{i}^{⊖}$ is the standard reaction free energy change of either the acid-base equilibrium reaction of the acid or the dissociation equilibrium reaction of the metal-ion pairs: *ANa*, or *ARb*. Here, Δ*v*_*i*_ corresponds to the difference in volume between the products and reactants. Thus, $\Delta G^{⊖}=\sum_{J}\nu_{J}\mu_{J}^{⊖}$ and Δ*v*_*i*_ = *∑*_*J*_ *ν*_*J*_ *v*_*J*_. Here *ν*_*J*_ is the stoichiometric coefficient of species *J* involved in either the acid-base or ion-condensation reaction. The variable $\beta \Delta G_{AH}^{sol}\left(r\right)$ is a position-dependent “solvation” free energy change and is given by

$$\Delta G_{AH}^{sol}\left(r\right)=\Delta u_{A^{-}}^{B}\left(r\right)+\Delta u_{H^{+}}^{B}\left(r\right)-\Delta u_{AH}^{B}\left(r\right)+E_{solv}\left(r\right)\left(v_{A^{-}}-v_{AH}\right)$$ (20)

$\Delta G_{ANa}^{solv}\left(r\right)$ and $\Delta G_{ARb}^{solv}\left(r\right)$ are defined in a similar fashion. Note, that the expression for $\Delta G_{i}^{sol}$ vanishes if a uniform dielectric background is assumed. In the above equation *E*_*solv*_(*r*) is given as

$$E_{solv}\left(r\right)=\frac{1}{2}\epsilon_{0}\epsilon_{r}^{\prime }\left[\phi_{PA}\left(r\right)\right]{\nabla_{r}\psi \left(r\right)}^{2}+\underset{k}{\sum }u_{k}^{B}\left(r\right)\rho_{k}\left(r\right)\frac{\epsilon_{r}^{\prime }\left[\phi_{PA}\left(r\right)\right]}{\epsilon_{r}\left[\phi_{PA}\left(r\right)\right]}.$$ (21)

with $\epsilon_{r}^{\prime }\left[\phi_{PA}\left(r\right)\right]$ corresponding to the functional derivative with respect to the volume fraction of the PA-nanofiber and $u_{k}^{B}\left(r\right)=z_{k}^{2}e^{2}/8\pi \epsilon_{0}\epsilon_{r}\left(r\right)a_{k}$ , i.e., to “absolute” electrostatic solvation energy. In our previously studies of weak polyelectrolyte layers, this solvation energy contribution did not appear since we only had considered the acid-base equilibrium, for which we assume $v_{A^{-}}=v_{AH}$ . The ion condensation has a contribution similar to Eq. 20, with a non-zero volume change equal to $\left(v_{A^{-}}-v_{ARb}\right)$ .

The $\Delta G_{i}^{⊖}$ is related to the chemical equilibrium constant, namely $K_{i}^{⊖}=\exp\left(-\beta \Delta G_{i}^{⊖}\right)$ . Explicitly, the standard free energy change of the acid-base equilibrium *AH ⇌ A* ^−^ + *H* ^+^ is given by $\Delta G_{AH}^{⊖}=\mu_{A^{-}}^{⊖}+\mu_{H^{+}}^{⊖}-\mu_{AH}^{⊖}$ , and the change in volume is equal to $\Delta v_{AH}=v_{A^{-}}+v_{H^{+}}-v_{AH}$ . The chemical equilibrium constant $K_{AH}^{⊖}$ is related to the experimental acid-base equilibrium constant $K_{AH}=C\exp\left(-\beta \Delta G_{AH}^{⊖}\right)$ of a single acidic monomer in infinitely dilute solution. Here *C* is a constant required for consistency of units and equal to *C* = 1/(*N*_*A*_ *v*_*w*_), where *N*_*A*_ is Avogadro’s number. This constant can be obtained readily by recasting the above chemical reaction into

$$\frac{\left[A^{-}\left(r\right)\right]\left[H^{+}\left(r\right)\right]}{\left[AH\left(r\right)\right]}=\frac{K_{AH}^{⊖}}{N_{A}v_{w}}e^{-\beta \Delta G_{AH}^{solv}\left(r\right)}e^{-\beta \pi \left(r\right)\Delta v_{AH}},$$ (22)

and observing that, in the limit of infinite dilution *π* → 0 and *ψ* → 0, the two exponents vanish and the right-hand side becomes equal to the experimental equilibrium constant of a carboxylic acid monomer in solution. The chemical equilibrium constant for the ion condensation reactions are obtained in an identical fashion. Inspection of above reaction equations reveals that with increasing lateral osmotic pressure, *π*(*r*), corresponds to decreasing volume fraction of water or increasing peptide amphiphile volume fraction, and negative electrostatic potential the acid-base equilibrium shifts towards the neutral state. A similar trend occurs with decreasing dielectric constant (increasing ⟨*ϕ*_*PA*_(*r*)⟩), in which the acid-base equilibrium shifts towards the neutral state.

To obtain a solution we need to solve the Poisson equation and incompressibility constraint simultaneously, since the unknowns in Eqs. 14, 15, 17, 18, 19 are the Lagrange multipliers or lateral pressures, *π*(*r*), and the electrostatic potential, *ψ*(*r*). Once the lateral pressures and the electrostatic potential are established, the amount of protonation and ion condensation of the Glu acids as well as the density distribution of the ions and the solvent are known. Solutions can be obtained by substituting the expressions of the volume fractions of all components into the incompressibility constraint Eq. 11 and the Poisson equation Eq. 16. This results in a set of non-linear integrodifferential equations that are converted into a set of coupled non-linear algebraic equations by discretizing space and these non-linear equations can be solved numerically (Hindmarsh et al., 2005). For detailed discretized expressions for individual terms and the computational procedure, the reader is referred to the supporting materials and previous publications (Nap et al., 2006; Nap et al., 2014a).

The inputs required to solve the non-linear equations are the concentrations of RbCl and NaCl, the reservoir *pH* the volume of all species, the volume fraction distribution of the PA-nanofiber and the distribution of chargeable sites of the Glu-residues. Also required are the acid-base equilibrium constants *pK*_*a*_ of the carboxylic acid and equilibrium dissociation constant of the Rb^+^ and Na^+^ ions with the carboxylate groups, all listed in the supporting information.

## 3 Results

Figure 1 shows a representative snapshot of the atomistic MD simulation of the peptide amphiphile self-assembled into a nanofiber structure. Here the alkyl chains comprise the core of the nanofiber and the water-exposed periphery is made up of the peptides. For clarify we also show the chemical structure of the peptide amphiphile. Atomistic molecular dynamics simulations were performed based on the previously equilibrated structures (Ortony et al., 2017). The simulation details are provided in the Supporting Materials. Shown in Figure 1C are the volume fraction distribution of the PA-fiber and the number density of the Glu units.

Using the above volume fraction profile and distribution of glutamic acid residues as input, we determine with the theory the amount of charge on the PA-nanofiber, the distribution of ions and water molecules and the electrostatic potential. To characterize the charging behavior of the PA-nanofiber and quantify the changes that occur due to changes in pH and salt concentration, we computed the average degree of dissociation. The average degree of dissociation ⟨*f*⟩ is obtained by integration of the local position-dependent degree of dissociation, $f_{A^{-}}\left(r\right)$ (Eq. 17), and given by

$$\left\langle f\right\rangle =\int drG\left(r\right)f_{A^{-}}\left(r\right)\rho_{Glu}\left(r\right)/\int drG\left(r\right)\rho_{Glu}\left(r\right).$$ (23)

**FIGURE 2:**
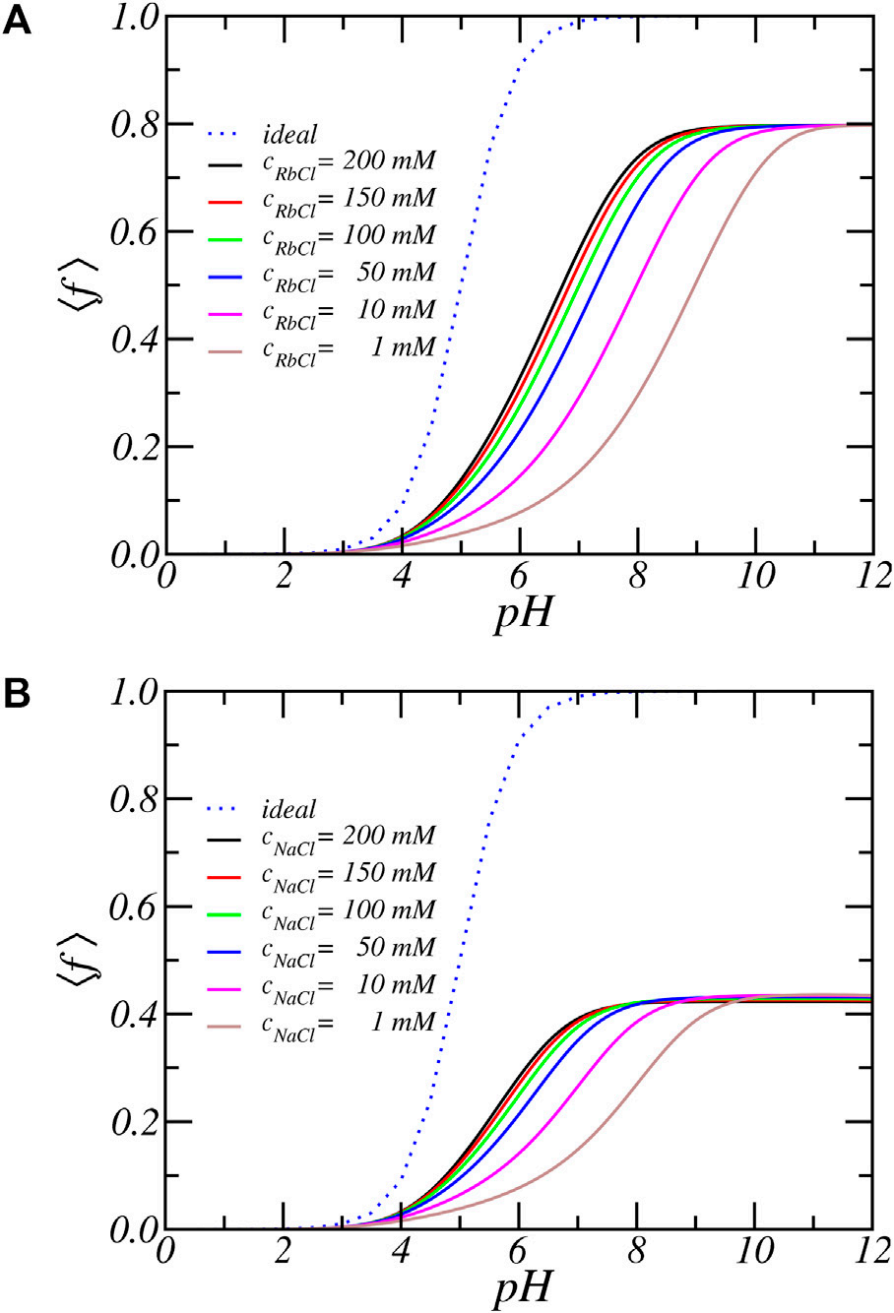
The average degree of dissociation as function of reservoir pH for increasing **(A)** RbCl and **(B)** NaCl salt concentrations. The line labeled ‘ideal’ represent the ideal solution behavior.

Figures 2A,B show the average degree of charge as a function of pH for PA-nanofiber immersed in aqueous solutions that have different concentrations of RbCl and NaCl, respectively. For comparison, the dotted line labelled ‘ideal’ shows the degree of charge of an isolated carboxylic acid molecule in dilute solution, which obeys the ideal solution chemical equilibrium equation: $\left\langle f\right\rangle =1/\left(1+10^{\left(pK_{a}-pH\right)}\right)$ .

There is a very large deviation from ideal solution behavior as well as a strong salt dependence. For all salt concentrations and pH values, the degree of dissociation of the acidic residues is significantly lower than predicted by ideal solution behavior. For example at *pH* = 7 a carboxylic acid in solution is almost completely charged (⟨*f*⟩ = 0.99), since its *pK*_*a*_ equals 5. But the same acid residue in the PA-nanofiber has a degree of deprotonation reduced to less than 50%. To be precise, ⟨*f*⟩ = 0.56 for [RbCl] = 200 *mM* and by decreasing the salt concentration to 1 *mM* the fraction of charged acids further reduces and becomes as low as 15.3%. Thus, the degree of charge as a function of pH or the titration curve shifts to much higher pH values. The apparent *pK*_*a*_, that is the pH for which half of the acid units are charged is shifted upwards by as much as 2–4 pH units, depending on the salt concentration. Also, we observe the occurrence of a plateau value of ⟨*f*⟩ = 0.8 at higher pH values. The fraction of charged acids never reaches 1. Thus, the amino acids inside a PA-nanofiber behave quantitatively and qualitatively differently from an isolated acid group.

**FIGURE 3:**
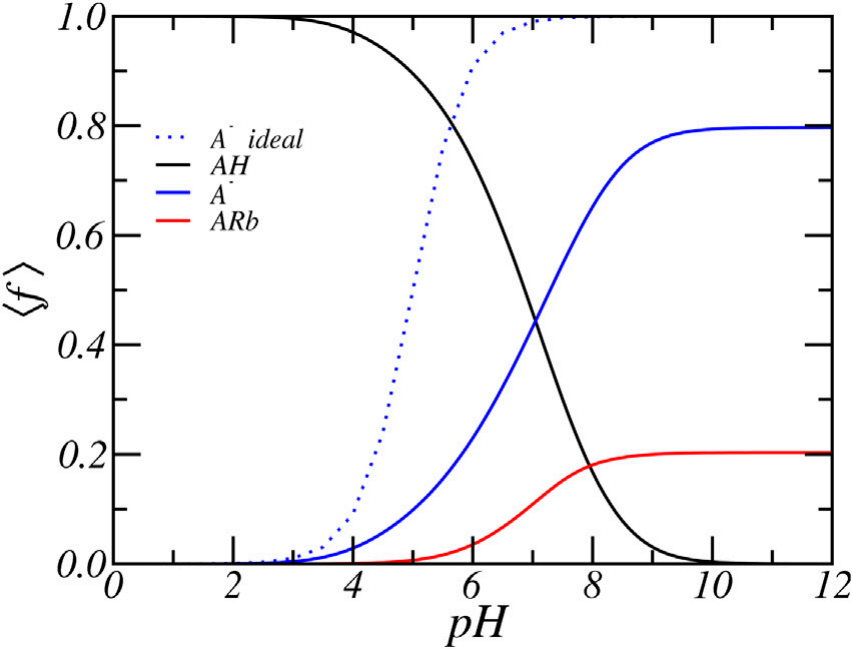
The average degree of protonation, deprotonation and ion-condensation of the glutamic acid residue as function of reservoir pH for a concentration of [RbCl] = 50 *mM*.

To explain the predicted trends, we first focus upon the case of relatively low concentration of [RbCl] = 50 *mM* and present in Figure 3 the average fraction of acids that are protonated, deprotonated, and condensed with Rb^+^ as a function of pH. At low pH, the acids are mostly found in their protonated state. With increasing pH, the acid starts to gradually deprotonate. Observe that significant deprotonation only occurs for pH values well above the *pK*_*a*_ of the carboxylic acid group. Simultaneously with deprotonation ions start to condense. The general behavior of the degree of dissociation as a function of salt concentration and pH can be understood as follows. The charge or degree of deprotonation of the amino acids occurs through a balance between the chemical free energy of the acid-base equilibrium reaction and the counterion ion condensation to the glutamic amino acids, the electrostatic interactions, and the mixing entropy of the solvent and mobile ions. To be exact, the entropy that is associated with counterion confinement. With decreasing salt concentrations, the electrostatic repulsion between the deprotonated amino acids are less screened and the system experiences increased electrostatic repulsion, for which the system tries to compensate. The PA-nanofiber system can respond in several ways.

First, the PA system recruits additional counterions from the reservoir, which increases the electrostatic screening. This decreases the (enthalpic) electrostatic repulsion at an entropic cost related to the loss of translational entropy of the counterions. Secondly, the electrostatic repulsion can be reduced by decreasing the number of deprotonated amino acids, which can be accomplished by either shifting the acid-base equilibria towards its protonated state or by condensing counterions. Both occur at the cost of their respective chemical free energies. For a weak acid the cost associated with the chemical free energy contribution is small as compared to the loss of entropy due to counterion confinement. Hence the mechanism of charge regulation through shifting the chemical equilibrium is usually the primary mode by which charged systems, such as weak polyelectrolytes and PA-nanofibers, try to mitigate the effects of electrostatic repulsion.

There is a third mechanism available to the peptide-amphiphiles to negate the electrostatic repulsion by increasing the intermolecular spacing between PA molecules. By changing the spatial distribution of the PA the charges are moved apart and the electrostatic repulsion decreases. Clearly, the approximation we use does not account for this possibility, since we assume a fixed spatial distribution of Glu residues within the PA-nanofibers. Observe that increasing the intermolecular spacing between PA molecules cannot negate nearest neighbor electrostatic interactions of charged Glu residues that reside on the same PA molecule. Also, the hydrophobic interaction of alkyl chains leads to the formation of the very dense core of the PA-nanofiber, this limits the possibility of increasing the intermolecular spacing between PA molecules within a (cylindrical) nanofiber. The possibility of molecular reorganization depends also on the strength of the *β*-sheet hydrogen bonds of the peptides. Only by a large structural transition, which involves enthalpic and entropic penalties, from a cylindrical nanofiber to a spherical micelle can the electrostatic penalty be reduced (Israelachvili, 2011; Zaldivar et al., 2018; Zaldivar et al., 2019). This occurs experimentally for pH above 8. We have investigated the effect of different spatial distributions of PA molecules on the charge of the PA-nanofiber. The distributions are obtained from MD simulations with different aggregation numbers. Calculations using different distributions resulted in qualitatively similar titration curves. We conclude that changes in spatial reorganization of the PA-nanofiber have a minor effect in determining the overall charge of the PA-nanofiber.

### 3.1 Ion-Condensation Vs Protonation

**FIGURE 4:**
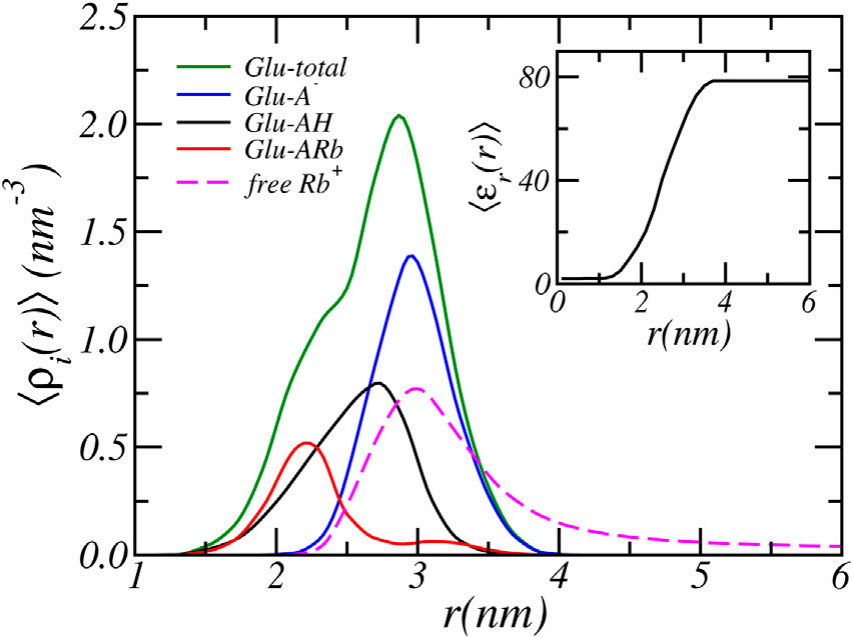
The radial cylindrical number density distribution of the total, and the protonated, deprotonated and Rb^+^ condensed Glu acid residues of the PA-nanofiber. For a reservoir pH of 7.4 and a RbCl concentration of [RbCl] = 50 *mM*. The inset shows the relative dielectric constant.

In the previous section we analyzed the charge regulation of a PA-nanofiber by considering the average degree of (de)protonation and ion condensed states of the Glu residues only. Here, to acquire further insight into the charging behavior of PA-nanofibers, we present in Figure 4 the position-dependent radial distribution of the protonated, deprotonated, and Rb^+^ condensed Glu acid residues alongside of the distribution of all Glu acid residue. We consider a physiological *pH* = 7.4 and an RbCl concentration of [RbCl] = 50 *mM*. The distribution shows that most deprotonated acids reside at the surface of the PA-nanofiber, where the carboxylic acids are most exposed to the water surrounding the PA-nanofiber. In contrast, the acids that are less exposed and “buried” inside the PA-nanofiber are either protonated or are condensed with Rb^+^ ions.

The relative dielectric constant is the volume weighted average of the dielectric constant of water (78.54) and the PA-nanofiber (2). See Eq. 10. Thus the dielectric constant is proportional to the PA-nanofiber volume fraction distribution as presented in Figure 1 and the dielectric constant smoothly transition from a value of 78.54 at the periphery to approximately two within the alkyl core. Therefore, acids that are located within the nanofiber experience a much lower dielectric environment. The dielectric constant as function of radial distance is shown in the inset of Figure 4. With decreasing dielectric constant the strength of electrostatic interactions increases. Simultaneously, with decreasing dielectric constant the free energy required to solvate charged, deprotonated acids and ions increases. Observe that this electrostatic solvation energy or Born energy is inversely proportional to the dielectric constant. Therefore, it is energetically more unfavorable for a “buried” acid to be deprotonated. In contrast, deprotonated acids are mostly found in the solvent-exposed terminus or surface of the PA-nanofiber which contains more water and has a higher dielectric constant compared to the interior of the PA-nanofiber.

Deprotonation of the terminal carboxylic acids near the surface of the PA-nanofiber is more favorable than for acids located in the interior of the PA-nanofiber. On the surface of the PA-nanofiber, the resulting excess electrostatic repulsion is mitigated by counterion confinement. Here, counterion confinement is energetically less expensive than completely shifting the chemical equilibrium to the neutral state or condensing ions. The dielectric environment is closer to that of water. Thus there is a smaller energetic penalty (in terms of solvation) for ions to penetrate the terminal part of the PA-nanofiber. It is still unfavorable, because of the loss of translational entropy due to ion confinement. By confining counterions in the solvent exposed region of nanofiber the acids remain deprotonated to a higher degree. Locating counterions within the denser part of the nanofiber is energetically far less unfavorable, because of the higher solvation energy because the dielectric constant is lower. Hence the concentration of counterions drops with the denser part of the PA-nanofiber. Thus within the denser part of the nanofiber electrostatic repulsion due to deprotonation can not be reduced by counterion confinement. Instead the electrostatic repulsions are avoided by reduction of the number of charged acids by shifting the acid-base equilibrium to the protonated state and by counterion condensation. Consequently, ions will only penetrate the surface region of the PA-nanofiber. This is clearly illustrated in Figure 4 where the dashed line corresponds to the distribution of free Rb^+^ ions. The free Rb^+^ ions are located outside and in the solvent exposed surface region of the PA-nanofiber where they overlap only with the distribution of deprotonated acids. Inside the PA-nanofiber, the distribution of free ions drops to zero. At the center of the PA-nanofiber, there are almost no solvents molecules present since the PA-nanofiber volume fraction approaches one and consequently the relative dielectric constant reaches a value of approximately two. There the Rb^+^ number density is very low (≲ 10^−40^ *nm* ^−3^), thus there are effectively no Rb^+^ ions located in the center of the fiber. This is analogous to the preference for ions to partition into water rather than an oil phase with a low dielectric constant.

A second interesting feature of Figure 4 is the position-dependent charge neutralization. We find that acids that are closest to the center of the PA-nanofiber have comparable amounts of ion-condensation and protonation, while acids located slightly further away from the center exhibit more protonation than ion condensation. Within increasing lower dielectric environments ion-condensation becomes relative more favorable compared to protonation.

**FIGURE 5:**
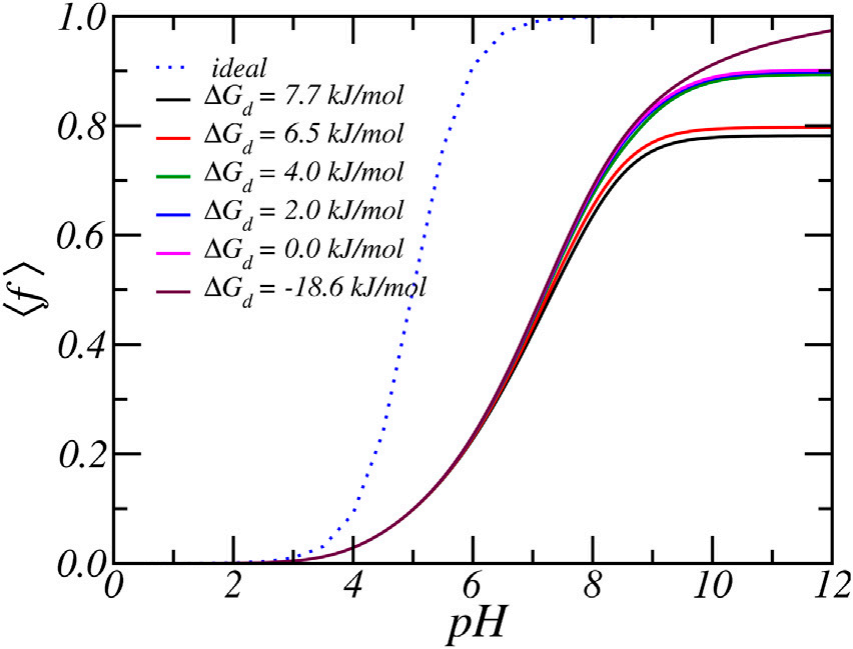
The average degree of protonation, as a function of reservoir pH for various carboxylate-Rb dissociation free energies. Form top to bottom the dissociation free energy, or equivalent the binding free energy decreases. The reservoir has a RbCl concentration of [RbCl] = 50 *mM*.

**FIGURE 6:**
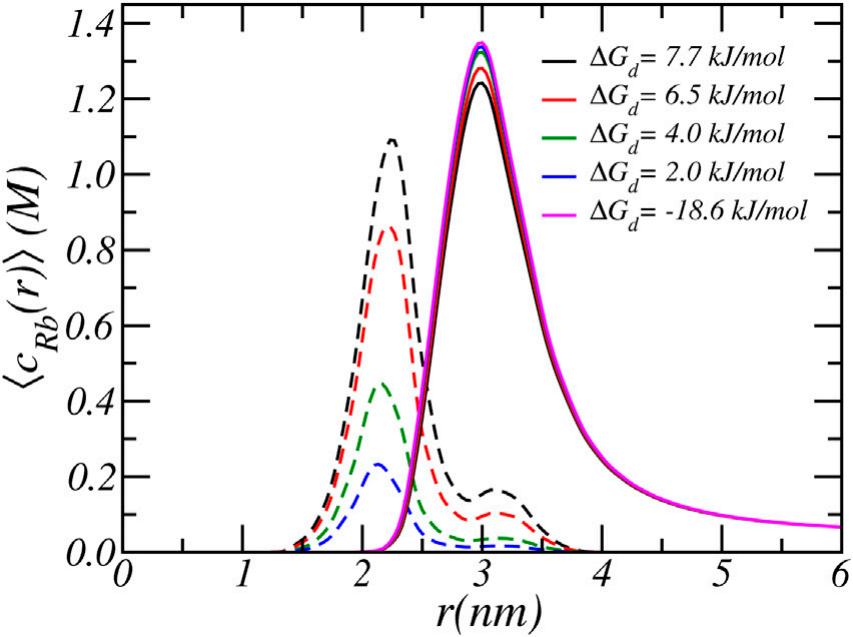
The radial cylindrical number density distribution of free Rb^+^ ions (solid lines) and bound Rb^+^ ions (dashed lines). The different lines correspond to different carboxylate-Rb dissociation free energies. The reservoir has a pH of 7.4 and a RbCl concentration of [RbCl] = 50 *mM*.

As discussed in the previous paragraphs, the equilibrium charged state of the Glu residue arises from a delicate interplay between various chemical and physical forces: include acid-base equilibrium and ion-condensation electrostatic and osmotic interactions. The balance between shifting the acid-base equilibrium and ion-condensation is also influenced by the strength of ion-binding (Δ*G*_*d*,*Rb*_) and the free energy associated with protonation (Δ*G*_*a*_). The latter value is known and directly related to the value of equilibrium constant, which is equal to *pK*_*a*_ = 5. On the other hand, the value of free energy of ion-binding is not well established. From past MD simulations, we found that the ion condensation of Na^+^ with acetate (i.e., carboxylic acid) is 7.7 *kJ*/*mol* (Park et al., 2018). Taking into account that Rb^+^ is larger than Na^+^, the free energy of ion binding of Rb^+^ must be smaller and we estimate a value of around 6.5 *kJ*/*mol*. To investigate the effect of the size of the free energy of ion condensation, we varied the value of the Rb-binding constant. Figures 5, 6 show the effect of variation of ion binding on the degree of deprotonantion and distribution of Rb^+^ ions. Unsurprisingly, with decreasing binding free energy the fraction of condensed ions is reduced. More interestingly, around physiological pH this decrease in ion condensation due to the reduction in the binding free energy does not result in a significant increase in the amount of deprotonation. The value of the binding free energy only influences the extent of deprotonation when the pH is significantly higher than physiological values.

At higher pH the acid-base equilibrium tends to shift to complete deprotonation and through ion condensation the resulting excess electrostatic repulsions can be mitigated. Consequently at higher pH values ion-condensation becomes the “primary” mode of charge regulation. This also explains the occurrence of the plateau value as observed in the titration curves. For a pH around physiological conditions, the total charge does not change when the binding energy is varied and the amount of deprotonation remains roughly the same. With increasing binding energy the amount of counterion condensation increases but this accompanied with an decrease in the amount of protonation. This effect can indirectly be observed in Figure 6, which shows the density of total and bound Rb^+^ ions for different binding constants. As the binding energy reduces the number of ions that are condensed reduces. However, the number of free Rb^+^ ions is not affected since the amount of charged acids is very similar. Thus, the number of confined counterions ions remains very similar. Note that the case of Δ*G*_*d*_ = − 18 *kJ*/*mol* corresponding to *pK*_*d*_ = − 5 has virtually no condensed ions.

Finally, observe that the titration curves for PA-nanofiber in a solution containing Na^+^ counterions have a much lower plateau value as compared to solutions containing Rb^+^ ions. This is a direct conquence of the fact that Na^+^ ions have a larger binding energy then Rb^+^ ions and electrostatic solvation energy since Na^+^ is smaller then Rb^+^. Thus although PA-nanofibers respond qualitatively similar to different ion species, quantitatively the charging behavior of PA nanofibers in different electrolyte solutions can be very different.

### 3.2 Effect of Dielectric Environment

In the previous sections, we predicted the charge of PA-nanofiber that included explicitly a varying position-dependent dielectric constant and electrostatic solvation energy. However, most theories describing electrolyte solutions and end-tethered polyelectrolytes layers assume a constant dielectric background. Hence, we shall explore in this section the necessity of employing a variable position-dependent dielectric constant and electrostatic solvation energy. We will explore the effect of the dielectric media by comparing results for different assumptions about the dielectric environment.

**FIGURE 7:**
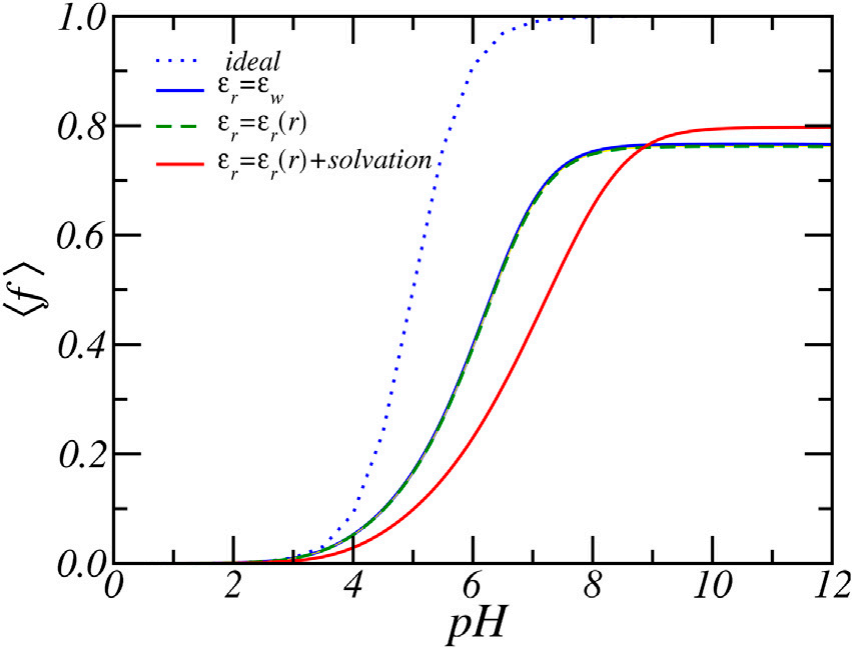
The average degree of charge as function of reservoir pH for fixed dielectric constant, varying dielectric constant and varying dielectric constant plus electrostatic self-energy. The reservoir has a RbCl concentration of [RbCl] = 50 mM.

Figure 7 shows the average degree of charge as a function of pH for different assumptions for the dielectric function. We assumed 1) a fixed dielectric constant (*ϵ*_*r*_(*r*) = *ϵ*_*w*_), 2) a varying dielectric constant and solvation energy, and 3) the case of varying dielectric constant with a fixed solvation energy. For the case of fixed dielectric constant, the relative dielectric constant is set equal to that of the water *ϵ*_*r*_ = *ϵ*_*w*_ = 78.54. The assumption of a fixed dielectric constant is a common approximation employed in theoretical studies of electrolyte and polyelectrolyte solutions (Ninham and Parsegian, 1971; Israelachvili, 2011). It is also commonly employed in theoretically studies of end-tethered (weak) polyelectrolyte layers (Israëls et al., 1994; Zhulina et al., 1995; Zhulina and Borisov, 2011). Here, even for comparatively dense weak polyelectrolyte layers, the charging behavior was similar for either a fixed dielectric constant or a varying dielectric function and electrostatic solvation energy (Nap et al., 2006; Nap et al., 2014b). Only for dense brushes at high salt concentration were significant changes found (Léonforte et al., 2016). Thus, assuming a fixed dielectric constant or a varying dielectric function resulted in structural and thermodynamics properties of end-tethered weak polyelectrolytes that are quantitatively and qualitatively very similar (Nap et al., 2014b). We found here that changes in the dielectric constant resulted in very large qualitative and quantitative differences in the PA nanofibers.

**FIGURE 8:**
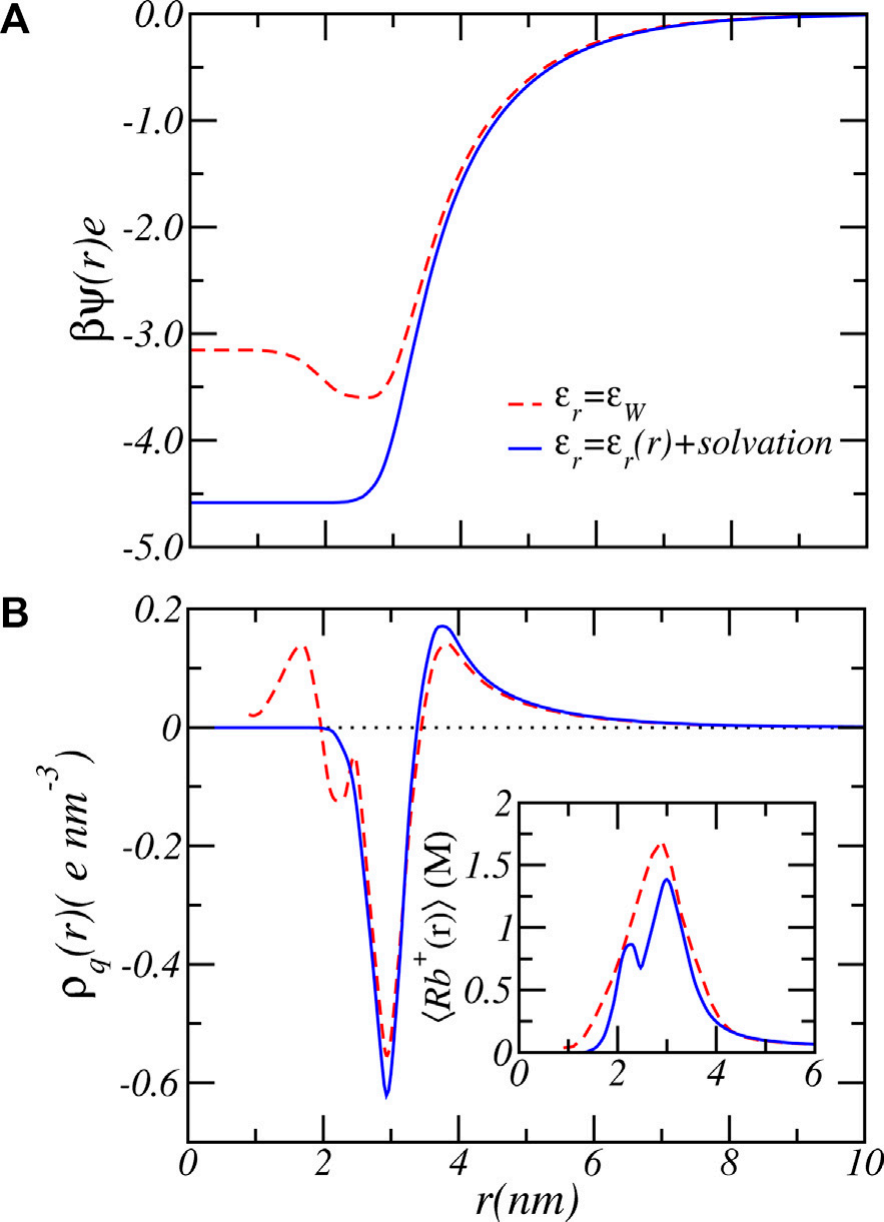
The electrostatic potential **(A)** and total charge number density **(B)** as function of radial coordinate for fixed dielectric constant (dashed lines) and varying dielectric constant plus electrostatic solvation energy (solid lines). The inset shows the total Rb^+^ ion concentration including free and bound ions. The reservoir has a pH value of 7.4 and a RbCl concentration of [RbCl] = 50 mM.

The results presented in Figure 7 clearly shows a large quantitative difference in the degree of charge that is most prominent around physiological pH values. For example, at *pH* = 7 bulk solution behavior would favor almost complete deprotonation with ⟨*f*⟩ = 0.99. Consideration of charge regulation assuming a fixed dielectric constant reduces the average degree of charge to ⟨*f*⟩ = 0.66 while a varying dielectric constant combined with the electrostatic solvation energy results in an average degree of charge of ⟨*f*⟩ = 0.43, which is 154% lower in the amount of charge. The dashed curve in Figure 7 shows the case of a varying dielectric constant but does not include a varying solvation energy. The results are almost identical to the case of *ϵ*_*r*_(*r*) = *ϵ*_*w*_, which indicates that the influence of the dielectric environment on the charge regulation is most strongly manifested via the indirect coupling with the solvation energy, a result in line with past calculations on end-tethered weak polyelectrolytes (Nap et al., 2006; Nap et al., 2014b). There is also a large qualitative difference between assuming a fixed dielectric constant and a varying dielectric constant and solvation energy, as demonstrated by Figure 8, which shows the electrostatic potential as a function of radial distance from the center of the nanofiber for both fixed dielectric constant and varying dielectric constant. The electrostatic potential, shown for the reservoir condition of *pH* = 7.4 and Rb^+^ salt concentration of [RbCl] = 50*mM*, differ both in size and shape. Increasing the pH to a higher value increases the difference even further. The difference can become almost a factor of two. See supporting material. A fixed dielectric constant results in a non-monotonic variation of the electrostatic potential, while a varying dielectric function and varying electrostatic solvation energy lead to a shape that monotonically decreases as a function of distance from the center of the PA-fiber. The difference is also reflected in the distribution of the total charge as shown in Figure 8B. Because of the large electrostatic potential, counterions will accumulate around the negatively charged carboxylate groups. Assuming a fixed dielectric constant implies that there is no electrostatic solvation energy penalty for ions to penetrate the PA-nanofiber. Hence the total charge distribution displays two positive peaks. Phrased alternatively, an electric double layer potential is set up on “both sides” of the negatively charged glutamic acid distribution. This will result in the observed non-monotonic behavior of the electrostatic potential for fixed dielectric constant. This behavior is clearly “unphysical” since ions have a low propensity to be located in regions that have low dielectric constant, from which they are expelled.

Finally, the dielectric function has also a significant influence on the size and shape of the counterion profile. Comparison of the ion density profiles between the case of *ϵ*_*r*_(*r*) = *ϵ*_*w*_ and varying *ϵ*_*r*_(*r*) and solvation energy, shows an a large decrease in their maximum value. Also in the amount counterions confined and bound by the PA-nanofiber is much reduced by considering a varying dielectric constant and electrostatic solvation energy instead of a fixed dielectric constant. The distribution of the Rb^+^ ions is important since the Rb^+^ counterion distribution can be probed in ASAXS or via ion counting experiments. Assuming *ϵ*_*r*_(*r*) = *ϵ*_*w*_ would result in qualitative and quantitatively different predictions.

## 4 Summary and Concluding Remarks

We have developed a theoretical model to describe and predict the charges found in self-assembled peptide amphiphile nanofibers as a function of pH and ion concentration. In particular, we computed the amount of charge on a nanofiber made of peptide amphiphiles with the sequence *C*_16_ − *V*_2_ *A*_2_ *E*_2_. Theoretically, we accounted for the acid-base equilibrium as well as the local dielectric environment by allowing for a position-dependent dielectric constant as well as a local solvation energy of the charged species. We find that the charge on the glutamic acid residues is much lower compared to the same acid in dilute solution. Surprisingly there is a very strong coupling between the acid-base equilibrium and the local dielectric environment. Considering a constant dielectric background instead of varying dielectric media results in qualitative very different degrees of deprotonation. Also, the shape and value of the electrostatic potential and counterion ion distribution are quantitatively and qualitatively different. Considering a constant dielectric media, a commonly applied approximation, result in counterion penetration into the alkyl region of the peptide amphiphile nanofiber. This erroneous result indicates that it is necessary to include a dielectric constant plus electrostatic solvation to properly describe the charging behavior of peptide amphiphiles.

We also considered the effect of counterion ion binding of Rb^+^ and Na^+^ to the carboxylate group of glutamic acid. We demonstrated that ion condensation can result in a considerable amount of bound ions, particularly for buried carboxylic groups. Counterion binding offers an additional mode of charge regulation and, depending on the strength of the metal-acid binding, a “bimodal” distribution of counterions is found with spatially distinct regions of bound and free of counterions. Experimentally, this counterion distribution could be revealed by X-ray scattering experiments.

It can potentially also be probed through nuclear magnetic resonance (NMR) measurements or inductively coupled plasma mass spectroscopy (ICPMS). NMR has been used to measure the macromolecule-bound metal ion concentrations in for example protein and DNA metal complexes (Kozlyuk et al., 2016) while ICPMS has been used to reveal the concentration of counterions located around double-stranded DNA (Gebala et al., 2015). Observe that the latter method measure the total amount of counterions and can not distinguish between condensed and free counterions.

While the theory presented here includes many important details related to charge regulation and electrostatic solvation is still a mean-field approach and as such does not include electrostatic fluctuations. Another approximation is that we imposed the PA volume fraction and glutamic acid density distribution. Similar approximations have been used to investigate DNA-coated nanoparticles (Kewalramani et al., 2013). This assumption can be rationalized by noting that the PA-nanofiber structure is compact and dense, which is particularly true for the aliphatic core of the fiber. Because of this approximation, we can only predict the charge of cylindrical PA-nanofibers and cannot investigate the transition to for example spherical micelles. Experimentally such transitions are known to occur for elevated pH values, roughly above pH 8. Hence, the predictions are only representative for cylindrical nanofibers up to pH 8. We also investigated the charge of PA-nanofibers that had a different PA-volume fraction and Glutamic number density distribution. The distributions are obtained from MD simulations of PA-nanofibers with different aggregation numbers, which provides qualitatively similar results for the charging behavior of the PA-nanofiber. Therefore, we believe that the trends presented here are relevant and that changes in the PA distribution will have only a minor effect on the charging of PA-nanofibers. However, it is important to emphasize that because the model assumes a static peptide nanofiber, the results presented here are qualitative. The PA distribution and the amount of charge on the glumatic acid residues are coupled together and can therefore influence each other. This interplay between structure and charge of the PA molecules is missing since the PA molecular distribution is assumed to be static and cannot respond to changes in the charge distribution. For future directions, we will add the conformational entropy of the PA-molecules into the theory and investigate how the charging of PA-molecules couples with the self-assemble of the PA-molecules.

Another limitation of the theoretical approach is that the local dielectric constant is taken to be the linear volume-weighted average of the dielectric constant of water and the PA-nanofiber (Eq. 10). Such a constitutive equation provides an empirical description of the dielectric properties of the system. However, the local dielectric constant is coupled with the local polarization of the molecules, which is in turn determined by the local density composition and the local electric field. The description of the dielectric properties of the PA nanofiber could be improved upon by considering a polarizable molecular model. An example of such a model can be found in reference (Nakamura et al., 2012).

Very noteworthy are the recent theoretical investigations by Zaldivar et al., who applied the same Molecular Theory approach to self-assemble of peptide amphiphiles and demonstrated the structural transitions between bilayer, cylindrical, and spherical structured nanofibers can occur as a function of pH (Zaldivar et al., 2018; Zaldivar et al., 2019). Observe they opted to represent the peptide amphiphiles with much more coarse-graining. This much more coarse-grained representation, based up the Martini-force field, of the peptide amphiphiles enabled the successful solving of the equations. Another important difference was that their approach did not include the possibility of either a varying dielectric constant or position-dependent solvation energy. However, our results demonstrate that the inclusion of varying dielectric constant and solvation energy leads to significant changes in the charge of cylindrical peptide amphiphiles nanofibers, which should influence the location or “phase” boundary of structural transitions. How the changes in charge of PA-nanofiber influence the location and the possibility of structural changes is difficult to access, since other nanostructures like spherical micelles will also undergo a similar charge regulation mechanism. The calculations presented here demonstrate that the amount of charge and thereby the electrostatic potential is sensitive to the dielectric environment and this effect needs to be considered.

Finally, note that the all-atom simulations we used to obtain an input of PA-nanofiber distribution assume that both glutamic acid residues are completely charged and are independent of environmental conditions like pH and salt concentration. The results from our theory here show that the charge of PA-nanofibers is not fixed and varies considerably, depending on the surrounding environment. This suggest that all-atom simulations should utilize grand-canonical reaction ensemble (Landsgesell et al., 2019) or constant pH ensemble (Donnini et al., 2011; Cote et al., 2014) for an more accurate description of the charging of the PA molecules, instead of conventional all-atom simulations that assume a fixed charge.

The results, here, indicate that to properly understand the charge on peptide amphiphiles we need to take into account both the capacity of charge regulation of the amino acid residues and its coupling with the local dielectric environment.

## Data Availability

The original contributions presented in the study are included in the article/Supplementary Material, further inquiries can be directed to the corresponding authors.
