# Supplementary material for: Acid-Base Equilibrium and Dielectric Environment Regulate Charge in Supramolecular Nanofibers
Source: Front Chem. 2022 Mar 16;10:852164. doi: 10.3389/fchem.2022.852164 (PMC8965714; doi:10.3389/fchem.2022.852164)
Supplement: Supplementary file 1 [file DataSheet1.PDF]

Supplementary Material

1 ATOMISTIC MOLECULAR DYNAMICS (MD) SIMULATIONS

All-atom explicit solvent MD simulations were performed using the package of GROMACS (version 2016.4) (Hess et al., 2008) The OPLS-AA force field (Jorgensen et al., 1996) was employed with the correction for long alkyl chains (L-OPLS force field (Siu et al., 2012)). The L-OPLS force field has demonstrated higher accuracy than the original OPLS-AA potential in reproducing a variety of experimental data for long hydrocarbon chains, for instance, density, heat of vaporization, gel-to-liquid-phase transition, chain conformation, diffusion coefficient, viscosity and solvation free energy. (Siu et al., 2012) Correspondingly, the recommended TIP3P-MOD water model (Sun and Kollman, 1995) was utilized, which has display good consistence regarding the solvation free energy of numerous long hydrocarbon organic molecules. (Siu et al., 2012) The structure of the water molecules was constrained via the SETTLE algorithm. (Miyamoto and Kollman, 1992) The L-OPLS + TIP3P-MOD potential has previously been employed by us to investigate amphiphilic surfactants in high valent, metal ions separation, which displayed good agreements with the experimental data. (Qiao et al., 2018, 2015, 2014) The original OPLS-AA force field parameters was employed for Na^+ ions.

The system consisted of 90 $C_{16} - V_2A_2E_2$ peptide amphiphile (PA) molecules. The initial structure of the PA nanofiber was obtained from the equilibrated configuration in our previous work (Ortony et al., 2017), where the united-atom GROMOS force field (Schmid et al., 2011) was employed. Nonpolar hydrogen atoms were added to generate the all-atom structure required for the OPLS-AA potential. 180 Na^+ counterions were added to neutralize the PA molecules. No extra salt was added. 41,838 water molecules were added to create the initial simulation box of 16*16*5.2 (X*Y*Z) nm^3. The three-dimensional periodic boundary conditions were applied. The system was first equilibrated using the steepest descent algorithm. It was further equilibrated for 10 ps at 298 K with the NTV ensemble (constant number of particles, temperature and volume). The isobaric-isothermal (NPT) ensemble was subsequently applied: an equilibration of 10 ps was carried out using the time step of 1 fs, which was followed by a 10 ps equilibration with the time step of 2 fs where all the covalent bonds involving hydrogen atoms were constrained.

In the production simulation, all the covalent bonds were constrained by means of the LINCS algorithm.Hess et al. (1997); Hess (2007) A time step of 2.5 fs was thus employed. The neighbor searching was calculated up to a distance of 1.3 nm and updated every 10 time steps. The Lennard–Jones 12-6 interaction energies were switched off from 1.1 nm to 1.3 nm. The Coulombic interactions were calculated up to 1.3 nm for the short-range contributions, with the long-range contributions calculated via the smooth particle mesh Ewald algorithm (PME). (Darden et al., 1993; Essmann et al., 1995) The temperatures of water molecules and solute molecules (peptide amphiphiles and counterions of Na^+) were separately coupled with the Nose–Hoover thermostat (reference temperature T = 298 K and characteristic time 0.5 ps). The semiisotropic Parrinello–Rahman barostat was posed with the reference pressure 1 bar, the characteristic time 4 ps, and the compressibility $4.5 \times 10^{-5}\, bar^{-1}$. The production simulation was run 200 ns. The convergence of the systems was justified by calculating the interaction energy between PA molecules and Na^+/water and the Z-dimensional simulation box length (Figure S1). The structure of the final simulation configuration is presented in Figure S2.

2 MOLECULAR THEORY

Here we present the total free energy describing self-assembling PA-molecules that includes also the conformational entropy as well

$$\frac{\beta W}{L} = \int \mathrm{d}rG(r)\rho_{PA}(r)\sum_{\alpha} P(\alpha,r)\ln P(\alpha,r) + \int \mathrm{d}rG(r)\rho_{PA}(r)(\ln(\rho_{PA}(r)v_w) - 1) \quad \text{(S1)}$$

$$+ \sum_{k=\{w,Na^+,Cl^-,Rb^+,H^+,OH^-\}} \int \mathrm{d}rA(r)\,\rho_k(r)(\ln\rho_k(r)v_w - 1 + \beta\mu_k^{\ominus}) \quad \text{(S2)}$$

$$+ \int \mathrm{d}rG(r)\langle\rho_{Glu}(r)\rangle\Big[f_{A^-}(r)\ln f_{A^-}(r) + f_{AH}(r)\ln f_{AH}(r) \quad \text{(S3)}$$

$$+ f_{ANa}(r)\ln f_{ANa}(r) + f_{ARb}(r)\ln f_{ARb}(r) \quad \text{(S4)}$$

$$+ f_{A^-}(r)\beta\mu_{A^-}^{\ominus} + f_{AH}(r)\beta\mu_{AH}^{\ominus} + f_{ANa}(r)\beta\mu_{ANa}^{\ominus} + f_{ARb}(r)\beta\mu_{ARb}^{\ominus}\Big] \quad \text{(S5)}$$

$$+ \beta\int \mathrm{d}rG(r)\left(\rho_q(r)\psi(r) - \frac{1}{2}\epsilon(r)(\nabla\psi(r))^2\right) + \sum_k \beta\int \mathrm{d}rA(r)u_k^B(r)\rho_k(r) \quad \text{(S6)}$$

$$+ \sum_{\alpha,\beta}\frac{\beta}{2}\int \mathrm{d}r\int \mathrm{d}r'\langle\rho_{PA,\alpha}(r)\rangle V_{\alpha,\beta}(r,r')\langle\rho_{PA,\beta}(r')\rangle \quad \text{(S7)}$$

$$+ \sum_{\gamma\in\{O,H\}}\int \mathrm{d}rG(r)\langle\rho_{PA,\gamma}(r)\rangle\Big[-f_\gamma(r)(\ln\langle\rho_{PA,\gamma}(r)\rangle v_w - 1) \quad \text{(S8)}$$

$$+ (1 - f_\gamma(r))\ln(1 - f_\gamma(r))\Big] + \int \mathrm{d}rG(r)\rho_{pair}(r)\Big[\ln\rho_{pair}(r)v_w - 1 + \beta\Delta G_{pair}\Big] \quad \text{(S9)}$$

$$+ \beta\int \mathrm{d}rG(r)\pi(r)\left[\langle\phi_{PA}(r)\rangle + \phi_w(r) + \sum_k \phi_k(r) - 1\right] \quad \text{(S10)}$$

$$+ \beta\int \mathrm{d}rG(r)\langle\rho_{PA,Glu}(r)\rangle\lambda(r)\,(f_{A^-}(r) + f_{AH}(r) + f_{ANa}(r) + f_{ARb}(r) - 1) \quad \text{(S11)}$$

$$+ \sum_{\gamma\in\{O,H\}}\int \mathrm{d}rG(r)\lambda_\gamma(r)\Big[\langle f_\gamma(r)\rho_{PA,\gamma}(r)\rangle - \rho_{pair}(r)\Big] \quad \text{(S12)}$$

$$- \sum_{k=\{Cl^-,OH^-\}}\beta\mu_k\int \mathrm{d}rG(r)\rho_k(r) \quad \text{(S13)}$$

$$- \beta\mu_{H^+}\int \mathrm{d}rG(r)\,\big(\rho_{H^+}(r) + f_{AH}(r)\langle\rho_{PA,Glu}(r)\rangle\big) \quad \text{(S14)}$$

$$- \beta\mu_{Na^+}\int \mathrm{d}rG(r)\,\big(\rho_{Na^+}(r) + f_{ANa}(r)\langle\rho_{PA,Glu}(r)\rangle\big) \quad \text{(S15)}$$

$$- \beta\mu_{Rb^+}\int \mathrm{d}rG(r)\,\big(\rho_{Rb^+}(r) + f_{ARb}(r)\langle\rho_{PA,Glu}(r)\rangle\big) \quad \text{(S16)}$$

$$+ \beta\mu_{PA}(\int \mathrm{d}rG(r)\langle\rho_{PA}(r)\rangle - N_{PA}/L) \quad \text{(S17)}$$

The first two terms (Eq. S1) describe the conformational and translational entropy of the PA molecules. Eq. S2 corresponds to the translational entropy and standard free energy of formation of the ions and solvent. The next seven terms correspond to the chemical free energy related to the acid-base equilibrium and ion-condensation reaction. Eqs S6 is the electrostatic energy and the electrostatic solvation energy. The seventh line describes the effective van der Waals interaction between the various residue of the PA molecule (amino acids and hydrocarbon chain). The eighth and ninth line described the ability of the PA molecules to form beta-sheets or hydrogen bonds. The last term describes the excluded volume interaction among all molecules of the system. The terms Eqs. S2 through S6 have been discussed in the main text and will not be further discussed here.

The first and second contributions of the free energy pertain to the conformational and translational entropy of the PA molecules. Here $\rho_{PA}(r)$ denotes the number density of the PA molecules that are located at position r. Since the PA molecule is a chain molecule it has spatial extent. Therefore the PA number density needs to be defined with respect to a particular unit of the PA molecule. For mathematical convenience, it is measured at the 'center' of the PA molecule, namely the hydrocarbon unit that links the peptide sesquence with the aliphatic tail. Other definitions like the first or last unit of the PA molecule can equally well be used. (Nap and Szleifer, 2013; Nap et al., 2013; Szleifer, 1997; Zaldivar et al., 2019, 2018) Given the number density of the PA chains at position r' we can compute the average number density of the PA molecules at location r that is given by:

$$\langle \rho_{PA}(r) \rangle = \int \mathrm{d}r' \frac{G(r')}{G(r)} \rho_{PA}(r') \sum_{\alpha} P(\alpha, r) n(\alpha; r, r'). \tag{S18}$$

Here, $P(\alpha, r)$ is the probability of finding a PA chain in conformation α that is located at position r. A conformation is given by a set of the positions of all monomers or unit of the PA chain. The probability distribution function or pdf is the central quantity in the molecular theory, since any thermodynamic and structural properties of the PA can be calculated from the probability distribution function, such as for instance, the average number density of the PA at position r. In Eq. S18 $n_{PA}(\alpha; r, r')\mathrm{d}r$ is the number of PA segments that a PA-chain in conformation α has within volume element $[r, r + dr]$. Observe, that the density of the chargeable unit of the glutamic acid residue is defined in a similar way, namely, $\langle \rho_{PA,Glu}(r) \rangle = \int \mathrm{d}r' \frac{G(r')}{G(r)} \sum_{\alpha} P(\alpha, r) n_{Glu}(\alpha; r, r')$, with $n_{Glu}(\alpha; r, r')\mathrm{d}r$ is the number of carboxylic unit of the Glutamic acid unit that the PA-chain in conformation α has within volume element $[r, r+dr]$. The next term in the free energy, Eq. S7 describes the non-excluded effective van der Waals attraction between the various units of the PA molecule which are denoted by $\langle \rho_{PA,\gamma}(r) \rangle$. Further details on the derivation of this contribution can be found in for example Refs. Szleifer and Carignano (1996); Tagliazucchi et al. (2010); Prusty et al. (2020).

The following contribution to the free energy, Eq. S9, describes the pairing free energy or hydrogen bonding free energy. It is modeled as a pairing between the oxygen of the carbonyl group of the amino acid unit of hydrogen linked with the nitrogen or amine group of the amino acid. The first term describes the entropic contribution and ΔG_{pair} is the enthalpic energy involved in the pair formation. Here $f_O(r)$ is the fraction number of oxygen of carbonyl the amino acid that forms a hydrogen bond and $f_H(r)$ is the fraction hydrogen that are linked of amine group of the amino acid that forms, a pair (or hydrogen bond). The variable $\rho_{pair(r)} = f_O(r) \langle \rho_{PA,O}(r) = f_H(r) \langle \rho_{PA,H}(r)$ equals the density of the number of pairs.

The following contribution in the free energy functional, Eq. S10 describes the steric repulsion among all molecules and is enforces through the introduction of the Lagrange multipliers. The next constraint, Eq. S11,

ensures that the fraction of state of the carboxylic acid add up to 1: $(f_{A^-}(r)+f_{AH}(r)+f_{ANa}(r)+f_{ARb}(r) = 1$. The Lagrange's multiplier in Eqs. S9 ensures $f_O(r)\langle\rho_{PA,O}(r) = f_H(r)\langle\rho_{PA,H}(r)$. The following terms Eqs. S13 through S16 describe the fact that the system is in equilibrium with a reservoir that contains ions of a given concentration or chemical potential. It also ensures particle conservation. The last contribution ensures that the aggregation number or number of PA molecules per unit length of the nanofiber equals N_{PA}/L.

Variation of the total free energy with respect to the electrostatic potential yields the Poisson Equation as presented in the main text. Likewise, minimization of the total free energy with respect to the ion densities, solvent, and the fraction of the charged states gives the equations presented in the main text. The total free energy present here also need to be minimized with respect to the PA number density ($\rho_{PA}(r)$), the probability distribution function ($P(\alpha, r)$), and $f_O(r)$ and $f_H(r)$.

Here to be brief, we only present the result for the PA number density and the probability distribution function in the absence of the van der Waals and pairing interactions. The probability distribution function is given by :

$$\begin{aligned} P(\alpha, r') &= \frac{1}{q(r)} \exp(-\beta \int \mathrm{d}r \pi(r) \sum_t n_t(\alpha, r'; r)) \\ &\times \exp(\beta \int \mathrm{d}r E_{solv}(r) \sum_t n_t(\alpha, r'; r)) \\ &\times \exp(-n_{Glu}(\alpha, r'; r) \left(\beta e \psi(r) + \ln(f_{A^-}(r))\right), \end{aligned} \tag{S19}$$

while the number density is given by

$$\rho_{PA}(r) = \frac{1}{v_w} e^{\mu_{PA}} q(r). \tag{S20}$$

Here $q(r)$ is the normalization or single-chain partition function that the pdf is properly normalized, while $E_{solve}(r)$ is the contribution associated with the varying dielectric constant and electrostatic solvation energy. The explicit form $E_{solve}(r)$ is given in Eq. (21) of the main text. In the case of a position-independent dielectric constant, $E_{solve}(r) = 0$, and the pdf become equal to the pdf presented earlier in for example Refs. (Nap et al., 2006, 2014). The equation for the pdf and the PA number density demonstrate that the configurational entropy is coupled with the local osmotic pressure $\pi(r)$ and electrostatic potential $psi(r)$. Changes in the charge result in changes in the electrostatic potential and local osmotic pressure, which result in in changes in the pdf that influence the distribution of the PA-molecule, which in turn influence the the electrostatic potential and local osmotic pressure. Further background on the molecular theory can be found in Refs. (Szleifer and Carignano, 1996; Nap et al., 2017; Solveyra et al., 2020; Nap et al., 2006). We are currently extending and implementing the above complete Molecular Theory.

2.1 Numerical methodology

Substituting the equations for solvent, ion and PA densities, Eqs. (14), (18) and (13) into the packing constraint (Eq.(11)), the generalized Poisson-Boltzmann equation (Eq. (16)), and the chemical reaction equations (Eqs. (17),(18), and (19)) results in a set of coupled integro-differential equations. The unknown of these equations are the position dependent lateral pressure $\pi(r)$, electrostatic potential $\psi(r)$ and total PA volume fraction $\langle\phi_{PA}(r)\rangle$. Observe a that we allow for volume change of the total PA volume fraction (see Eq. (13) of the main text): $\langle\phi_{PA}(r)\rangle = \langle\phi^0_{PA}(r)\rangle + \sum_{i\in\{AH,ARb,ANa\}}\langle\rho_{Glu}(r)\rangle f_i(r)(v_i - v_{A^-})$. Here

$\langle\phi^0_{PA}(r)\rangle$ and $\langle\rho_{Glu}(r)\rangle$ are given and input. If the chemical reactions would not result in volume changes, only lateral pressure $\pi(r)$ and electrostatic potential $\psi(r)$ would be unknowns. A numerical solution for the lateral pressure $\pi(r)$, electrostatic potential $\psi(r)$ and the total volume fraction $\langle phi_{PA}(r)\rangle$ is obtained by discretization of the packing constraints, Eq. (11), and the generalized Poisson Equation (Eq. (16)) and the chemical reactions equations (Eqs. (17),(18), and (19)) and the equation for the total volume fraction (Eq. (13)). The equations are discretized by dividing the r-coordinate into cylindrical shells of thickness δ. Position dependent functions are assumed to be constant within a cylindrical, hence integrations can be replaced by summations. The integral of a general position dependent function $f(r)$ then becomes:

$$\int_V \mathrm{d}r G(r)f(r) = \sum_i \int_{(i-1)\delta+R}^{i\delta+R} \mathrm{d}r\, G(r)f(r) \approx \sum_i f(i)\Delta G(i), \tag{S21}$$

with

$$\Delta G(i) = \int_{(i-1)\delta=R}^{i\delta+R} \mathrm{d}r\, G(r). \tag{S22}$$

Here $f(i)$ denotes the value which function $f(r)$ attains within the cylindrical region located between $(i-1)\delta \leq r < i\delta$ and $G(r)dr = 2\pi r dr$ corresponds to a volume element per unit length of the fiber. The geometric factor $\Delta G(i)$ is the finite volume of the discrete cylindrical shell.

The packing constraint, Eq. (13), in discrete form for grid cell (i) reads

$$\langle\phi_{PA}(i)\rangle + \phi_w(i) + \phi_{Na^+}(i) + \phi_{Cl^-}(i) + \phi_{Rb^+}(i) + \phi_{H^+}(i) + \phi_{OH^-}(i) = 1. \tag{S23}$$

The volume fraction of water, Eq. (18), the PA volume fraction, Eq. (13) in discrete form become

$$\begin{aligned} \phi_w(i) &= \exp(-\beta\pi(i)v_w), && \text{(S24)}\\ \langle\phi_{PA}(i)\rangle &= \langle\phi^0_{PA}(i)\rangle + \sum_{k\in\{AH,ARb,ANa\}} \langle\rho_{Glu}(i)\rangle f_k(i)(v_k - v_{A^-}). && \text{(S25)} \end{aligned}$$

The volume fraction of the counterion, coions, protons, and hydroxyl ions, (Eqs.(17), (18),and (19)), in discretized space are:

$$\begin{aligned} \phi_{Na^+}(i) &= \phi_{Na^+,bulk}\exp(-\beta(\pi(i)-\pi_{bulk})v_{Na^+} - e\beta\psi(i) - \beta\Delta u^B_{Na^+}(i)), && \text{(S26)}\\ \phi_{Rb^+}(i) &= \phi_{Rb^+,bulk}\exp(-\beta(\pi(i)-\pi_{bulk})v_{Rb^+} - e\beta\psi(i) - \beta\Delta u^B_{Rb^+}(i)), && \text{(S27)}\\ \phi_{Cl^-}(i) &= \phi_{Cl^-,bulk}\exp(-\beta(\pi(i)-\pi_{bulk})v_{Cl^-} + e\beta\psi(i) - \beta\Delta u^B_{Cl^-}(i)), && \text{(S28)}\\ \phi_{H^+}(i) &= \phi_{H^+,bulk}\exp(-\beta(\pi(i)-\pi_{bulk})v_{H^+} - e\beta\psi(i) - \beta\Delta u^B_{H^+}(i)), && \text{(S29)}\\ \phi_{OH^-}(i) &= \phi_{OH^-,bulk}\exp(-\beta(\pi(i)-\pi_{bulk})v_{OH^-} + e\beta\psi(i) - \beta\Delta u^B_{OH^-}(i)), && \text{(S30)} \end{aligned}$$

with $\Delta u^B_k(i)$ is the discretized electrostatic solvation energy:

$$\Delta u^B_k(i) = \frac{z_k^2 e^2}{8\pi\epsilon_0 a_k}\left(\frac{1}{\epsilon_r(i)} - \frac{1}{\epsilon_w}\right). \tag{S31}$$

Here $\epsilon_r(i)$ is the relative dielectric constant at cylindrical shell i,

$$\epsilon_r(i) = \epsilon_{PA}\langle\phi_{PA}(i)\rangle + \epsilon_w(1 - \langle\phi_{PA}(i)\rangle). \tag{S32}$$

The above volume fractions depend on the lateral pressure, electrostatic potential, and the bulk volume fractions. The chemical potentials of the counter and co-ions, protons, and hydroxyl ions are related to their bulk volume fractions. Nap et al. (2006) These bulk values are input to the theory.

The chemical reaction Eqs. ((17),(18), and (19)) are in discrete form Nap et al. (2014, 2018)

$$\frac{f_{A^-}(i)}{f_{AH}(i)} = K^{\ominus}_{AH}\frac{e^{-\beta\Delta G^{solv}_{AH}(i)}e^{-\beta\pi(i)\Delta v_{AH}}}{\rho_{H^+}(i)v_w}, \tag{S33}$$

$$\frac{f_{A^-}(i)}{f_{ANa}(i)} = K^{\ominus}_{ANa}\frac{e^{-\beta\Delta G^{solv}_{ANa}(i)}e^{-\beta\pi(i)\Delta v_{ANa}}}{\rho_{Na^+}(i)v_w} \tag{S34}$$

$$\frac{f_{A^-}(i)}{f_{ARb}(i)} = K^{\ominus}_{ARb}\frac{e^{-\beta\Delta G^{solv}_{ARb}(i)}e^{-\beta\pi(r)\Delta v_{ARb}}}{\rho_{Rb^+}(r)v_w}, \tag{S35}$$

$$1 = f_{A^-}(i) + f_{AH}(i) + f_{ANa}(i) + f_{ARb}(i), \tag{S36}$$

with

$$\Delta G^{sol}_{AM}(i) = \Delta u^B_{A^-}(i) + \Delta u^B_{M^+}(r) - \Delta u^B_{AM}(r) + E_{solv}(r)(v_{A^-} - v_{AM}), \tag{S37}$$

$$\text{with} \quad M = \{H, Na, Rb\}, \tag{S38}$$

$$E_{solv}(i) = \frac{1}{2}\epsilon_0\epsilon'_r[\phi_{PA}(i)]\nabla_r\psi(i)^2 + \sum_{k=\{\text{all charged species}\}} u^B_k(i)\rho_k(i)\frac{\epsilon'_r[\phi_{PA}(i)]}{\epsilon_r[\phi_{PA}(i)]}, \tag{S39}$$

$$\epsilon'_r[\phi_{PA}(i)] = \epsilon_{PA} - \epsilon_w. \tag{S40}$$

In above, the discrete nabla-operator is given by:

$$\nabla_r\psi(i) = \begin{cases} \frac{\psi(i+1)-\psi(i-1)}{2\delta} + \mathcal{O}(\delta^2) & i \neq i, \\ \frac{\psi(2)-\psi(0)}{2\delta} + \mathcal{O}(\delta^2) = \frac{\psi(2)-\psi(1)}{2\delta} + \mathcal{O}(\delta^2) & i = 1. \end{cases} \tag{S41}$$

Here we used notation $\psi(i) = \psi(r_i)$ with $r_i = (i - 1/2)\delta$ denoting the middle of the cylindrical cell (i). In above Eq. S41 we made used of the fact that the derivative at $r = 0$ is zero.

The discretized Poisson equation in cylindrical coordinates, is given

$$g_+(i)\frac{\epsilon(i+1)+\epsilon(i)}{2}\bar{\psi}(i+1) + \left[g_+(i)\frac{\epsilon(i+1)+\epsilon(i-1)}{2} + g_-(i)\frac{\epsilon(i)+\epsilon(i-1)}{2}\right]\bar{\psi}(i)$$

$$+ g_-(i)\frac{\epsilon(i)+\epsilon(i-1)}{2}\bar{\psi}(i+1) = -\left[\frac{\beta e^2\delta^2}{\epsilon_0\epsilon_w v_w}\right]\bar{\rho}_q(i) = c\bar{\rho}_q(i). \tag{S42}$$

Here we rescaled and made the electrostatic potential and charge number density dimensionless: $\bar{\psi} \stackrel{\text{def}}{=} \beta e\psi$ and $\bar{\rho}_q \stackrel{\text{def}}{=} v_w\rho_q/e$. Likewise the dielectric constant is rescaled :$\bar{\epsilon}(i) \stackrel{\text{def}}{=} \epsilon_r(i)/\epsilon_w$. To obtain above discretization scheme we used the finite volume method also refered to as box integration method. We assumed that there the dielectric constant at the edge of the cylindrical shells (i.e., the edges of integration volume) are given as a linear interpolation of the values in the middle of the adjetion cylindrical shells. $\epsilon(r_{i+1/2} = (\epsilon(r_{i+1} + \epsilon(r_i)/2$ In above equation $g_+(i)$ and $g_-(i)$ correspond to

$$g_+(i) \stackrel{\text{def}}{=} \frac{r_{i+1/2}}{r_i}, \quad \text{and} \quad g_-(i) \stackrel{\text{def}}{=} \frac{r_{i-1/2}}{r_i}. \tag{S43}$$

The Poisson equation for first (i=1) cylindrical shell reads:

$$g_+(1)\frac{\epsilon(2)+\epsilon(1)}{2}(\bar{\psi}(2)-\bar{\psi}(1)) = c\bar{\rho}_q(i). \tag{S44}$$

Substituting Eqs. (S24), through (S30) into the packing constraint equation (S23), the Poisson Equation (Eqs. (S42) and (S44)), and the chemical reaction equations Eqs. (S33) through (S36) in a set of coupled nonlinear algebraic equations which can be solved by standard numerical methods. Hindmarsh et al. (2005)

The inputs required to solve the non-linear equations are the concentrations of RbCl and NaCl, the reservoir pH the volume of all species, the volume fraction distribution of the PA-nanofiber, and the distribution of chargeable sites of the Glu-residues. Also required are the acid-base equilibrium constants $pK_a = 5$ of the carboxylic acid and equilibrium dissociation constant of the Rb^+ and Na^+ ions with the carboxylate groups, The ion-binding ($\Delta G_{d,Na}$) is $7.7kJ/mol$, obtained from MD simulation described in Ref. (Park et al., 2018). We estimate the ion-binding of Rb^+ with carboxylate and to be around $6.5kJ/mol$, as eloborated in the main text. Below in tables S1 we list the value of the volume ions used throughout the calculations. We use $\delta v_{AH} = v_w$ for the acid base equilibrium and set $\delta v_k = 0$ for all other reactions. We used a discretization length $\delta = 0.2\,nm$.

3 SUPPLEMENTARY TABLES AND FIGURES

3.1 Figures

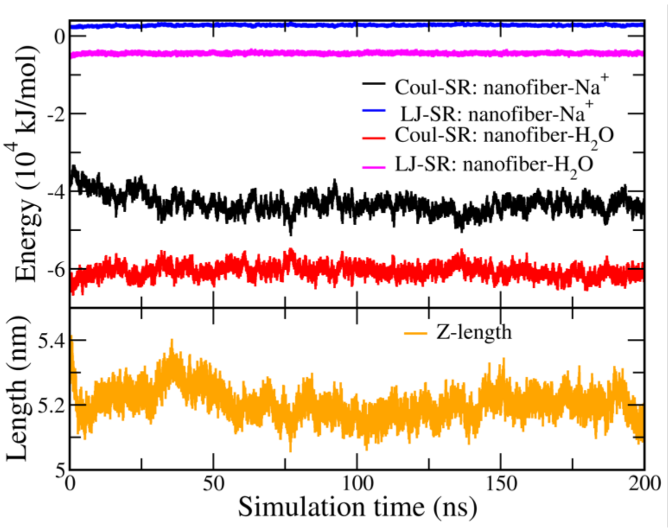

Figure S1. Convergence of the all-atom simulation. (Top) Short-range coulombic and Lennard-Jones interaction energy of nanofiber-Na$^+$ and nanofiber-H_2O as a function of the simulation time, where the calculations were performed up to the cutoff distance of 1.3 nm. (bottom) The simulation box Z-edge length as a function of the time.

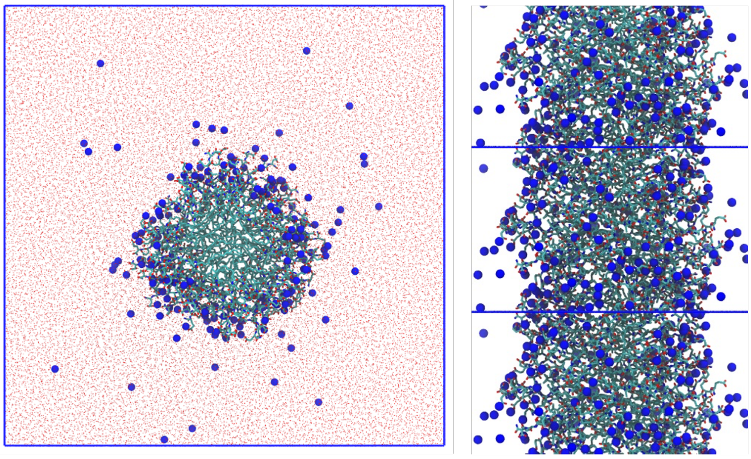

Figure S2. Snapshot of the last simulation configuration. (left) Top view. PA-nanofiber is highlighted at the center, where hydrogen atoms are omitted for display. Na$^+$ ions are highlighted by blue beads. Water molecules are colored red. The blue solid lines denote the boundary of the simulation box. (right) Side view of the PA nanofiber and Na$^+$ ions.

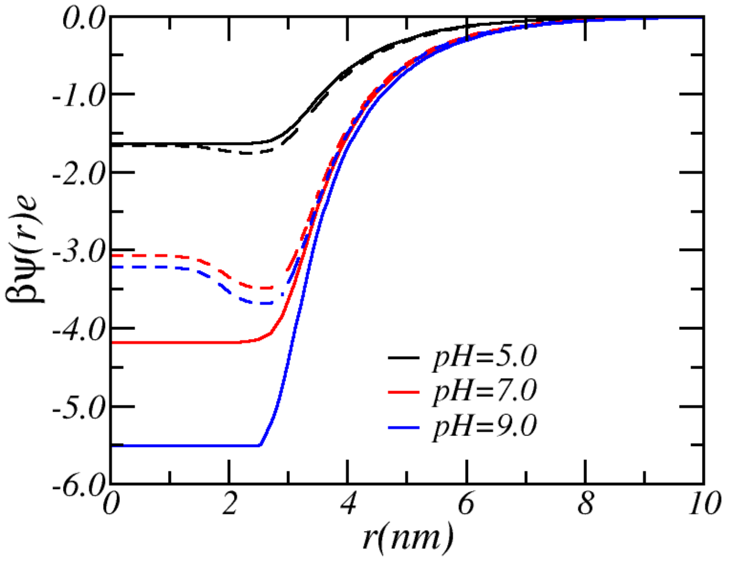

Figure S3. The electrostatic potential as function of radial coordinate for fixed dielectric constant (dashed lines) and varying dielectric constant plus electrostatic solvation energy (solid lines) for different reservoir pH values. The RbCl concentration of [RbCl]=50mM

3.2 Tables

Table S1. Volume of sovent and ions in nm^3.

	w	H^+	OH^-	Na^+	Cl^-	Rb^+
$v(nm^3)$	0.03	0.03	0.03	0.0044	0.0248	0.0147

REFERENCES

Darden, T., York, D., and Pedersen, L. (1993). Particle mesh ewald: AnN·log(n) method for ewald sums in large systems. *J. Chem. Phys.* 98, 10089–10092. doi:10.1063/1.464397

Essmann, U., Perera, L., Berkowitz, M. L., Darden, T., Lee, H., and Pedersen, L. G. (1995). A smooth particle mesh ewald method. *J. Chem. Phys.* 103, 8577–8593. doi:10.1063/1.470117

Hess, B. (2007). P-LINCS: a parallel linear constraint solver for molecular simulation. *J. Chem. Theory Comput.* 4, 116–122. doi:10.1021/ct700200b

Hess, B., Bekker, H., Berendsen, H. J. C., and Fraaije, J. G. E. M. (1997). LINCS: A linear constraint solver for molecular simulations. *J. Comput. Chem.* 18, 1463–1472. doi:10.1002/(sici)1096-987x(199709)18:12⟨1463::aid-jcc4⟩3.0.co;2-h

Hess, B., Kutzner, C., van der Spoel, D., and Lindahl, E. (2008). GROMACS 4: algorithms for highly efficient, load-balanced, and scalable molecular simulation. *J. Chem. Theory Comput.* 4, 435–447. doi:10.1021/ct700301q

Hindmarsh, A. C., Brown, P. N., Grant, K. E., Lee, S. L., Serban, R., Shumaker, D. E., et al. (2005). SUNDIALS: Suite of nonlinear and differential/algebraic equation solvers. *ACM Trans Math Software* 31, 363–396

Jorgensen, W. L., Maxwell, D. S., and Tirado-Rives, J. (1996). Development and testing of the opls all-atom force field on conformational energetics and properties of organic liquids. *J. Am. Chem. Soc.* 118, 11225–11236. doi:10.1021/ja9621760

Miyamoto, S. and Kollman, P. A. (1992). Settle: An analytical version of the SHAKE and RATTLE algorithm for rigid water models. *J. Comput. Chem.* 13, 952–962. doi:10.1002/jcc.540130805

Nap, R., Gong, P., and Szleifer, I. (2006). Weak polyelectrolytes tethered to surfaces: effect of geometry, acid-base equilibrium and electrical permittivity. *J. Polym. Sci., Part B: Polym. Phys.* 44, 2638–2662. doi:10.1002/polb.20896

Nap, R. J., Park, S. H., and Szleifer, I. (2018). Competitive calcium ion binding to end-tethered weak polyelectrolytes. *Soft Matter* doi:10.1039/C7SM02434G. In press

Nap, R. J., Park, Y., Wong, J. Y., and Szleifer, I. (2013). Adsorption of acid and polymer coated nanoparticles: a statistical thermodynamics approach. *Langmuir* 29, 14482–14493. doi:10.1021/la403143a

Nap, R. J. and Szleifer, I. (2013). How to optimize binding of coated nanoparticles: Coupling of physical interactions, molecular organization and chemical state. *Biomater. Sci.* 1, 814 – 823. doi:10.1039/c3bm00181d

Nap, R. J., Tagliazucchi, M., Gonzalez Solveyra, E., Ren, C.-l., Uline, M. J., Szleifer, I., et al. (2017). *Polymer and Biopolymer Brushes* (John Wiley & Sons, Inc.), chap. Modeling of Chemical Equilibria in Polymer and Polyelectrolyte Brushes. 161–221. doi:10.1002/9781119455042.ch6

Nap, R. J., Tagliazucchi, M., and Szleifer, I. (2014). Born energy, acid-base equilibrium, structure and interactions of end-grafted weak polyelectrolyte layers. *J. Chem. Phys.* 140, 024910. doi:10.1063/1.4861048

Ortony, J. H., Qiao, B., Newcomb, C. J., Keller, T. J., Palmer, L. C., Deiss-Yehiely, E., et al. (2017). Water dynamics from the surface to the interior of a supramolecular nanostructure. *J. Am. Chem. Soc.* 139, 8915–8921. doi:10.1021/jacs.7b02969

Park, S. H., Nap, R. J., and Szleifer, I. (2018). Association Free Energies of Metal Cations with Mesylate and Acetate in Brine Calculated via Molecular Dynamics Simulation. *ArXiv e-prints* , arXiv:1801.05888[physics.chem–ph]

Prusty, D., Nap, R. J., Szleifer, I., and de la Cruz, M. O. (2020). Charge regulation mechanism in end-tethered weak polyampholytes. *Soft Matter* 16, 8832–8847. doi:10.1039/d0sm01323d

Qiao, B., Demars, T., de la Cruz, M. O., and Ellis, R. J. (2014). How hydrogen bonds affect the growth of reverse micelles around coordinating metal ions. *J. Phys. Chem. Lett.* 5, 1440–1444. doi:10.1021/jz500495p

Qiao, B., Ferru, G., Olvera de la Cruz, M., and Ellis, R. J. (2015). Molecular origins of mesoscale ordering in a metalloamphiphile phase. *ACS Central Science* 1, 493–503. doi:10.1021/acscentsci.5b00306

Qiao, B., Littrell, K. C., and Ellis, R. J. (2018). Liquid worm-like and proto-micelles: water solubilization in amphiphile–oil solutions. *Phys. Chem. Chem. Phys.* 20, 12908–12915. doi:10.1039/c8cp00600h

Schmid, N., Eichenberger, A. P., Choutko, A., Riniker, S., Winger, M., Mark, A. E., et al. (2011). Definition and testing of the GROMOS force-field versions 54a7 and 54b7. *Eur. Biophys. J.* 40, 843–856. doi:10.1007/s00249-011-0700-9

Siu, S. W. I., Pluhackova, K., and Böckmann, R. A. (2012). Optimization of the OPLS-AA force field for long hydrocarbons. *J. Chem. Theory Comput.* 8, 1459–1470. doi:10.1021/ct200908r

Solveyra, E. G., Nap, R. J., Huang, K., and Szleifer, I. (2020). Theoretical modeling of chemical equilibrium in weak polyelectrolyte layers on curved nanosystems. *Polymers* 12, 2282. doi:10.3390/polym12102282

Sun, Y. and Kollman, P. A. (1995). Hydrophobic solvation of methane and nonbond parameters of the TIP3p water model. *J. Comput. Chem.* 16, 1164–1169. doi:10.1002/jcc.540160910

Szleifer, I. (1997). Protein adsorption on surfaces with grafted polymers: A theoretical approach. *Biophys. J.* 72, 595–612. doi:10.1016/S0006-3495(97)78698-3

Szleifer, I. and Carignano, M. A. (1996). Tethered polymer layers. *Adv. Chem. Phys.* 94, 165–260

Tagliazucchi, M., Olvera de la Cruz, M., and Szleifer, I. (2010). Self-organization of grafted polyelectrolyte layers via the coupling of chemical equilibrium and physical interactions. *Proc. Natl. Acad. Sci. U.S.A.* 107, 5300. doi:10.1073/pnas.0913340107

Zaldivar, G., Samad, M. B., Conda-Sheridan, M., and Tagliazucchi, M. (2018). Self-assembly of model short triblock amphiphiles in dilute solution. *Soft Matter* 14, 3171–3181. doi:10.1039/c8sm00096d

Zaldivar, G., Vemulapalli, S., Udumula, V., Conda-Sheridan, M., and Tagliazucchi, M. (2019). Self-assembled nanostructures of peptide amphiphiles: Charge regulation by size regulation. *J. Phys, Chem. C* 123, 17606–17615. doi:10.1021/acs.jpcc.9b04280
